# The Sail-Backed Reptile *Ctenosauriscus* from the Latest Early Triassic of Germany and the Timing and Biogeography of the Early Archosaur Radiation

**DOI:** 10.1371/journal.pone.0025693

**Published:** 2011-10-14

**Authors:** Richard J. Butler, Stephen L. Brusatte, Mike Reich, Sterling J. Nesbitt, Rainer R. Schoch, Jahn J. Hornung

**Affiliations:** 1 Bayerische Staatssammlung für Paläontologie und Geologie, München, Germany; 2 GeoBio-Center, Ludwig-Maximilians-Universität München, Munich, Germany; 3 Division of Paleontology, American Museum of Natural History, New York, New York, United States of America; 4 Department of Earth and Environmental Sciences, Columbia University, New York, New York, United States of America; 5 Museum, Sammlungen und Geopark, Geowissenschaftliches Zentrum, Georg-August-Universität Göttingen, Göttingen, Germany; 6 Department of Biology, University of Washington, Seattle, Washington, United States of America; 7 Staatliches Museum für Naturkunde, Stuttgart, Germany; Raymond M. Alf Museum of Paleontology, United States of America

## Abstract

**Background:**

Archosaurs (birds, crocodilians and their extinct relatives including dinosaurs) dominated Mesozoic continental ecosystems from the Late Triassic onwards, and still form a major component of modern ecosystems (>10,000 species). The earliest diverse archosaur faunal assemblages are known from the Middle Triassic (c. 244 Ma), implying that the archosaur radiation began in the Early Triassic (252.3–247.2 Ma). Understanding of this radiation is currently limited by the poor early fossil record of the group in terms of skeletal remains.

**Methodology/Principal Findings:**

We redescribe the anatomy and stratigraphic position of the type specimen of *Ctenosauriscus koeneni* (Huene), a sail-backed reptile from the Early Triassic (late Olenekian) Solling Formation of northern Germany that potentially represents the oldest known archosaur. We critically discuss previous biomechanical work on the ‘sail’ of *Ctenosauriscus*, which is formed by a series of elongated neural spines. In addition, we describe *Ctenosauriscus*-like postcranial material from the earliest Middle Triassic (early Anisian) Röt Formation of Waldhaus, southwestern Germany. Finally, we review the spatial and temporal distribution of the earliest archosaur fossils and their implications for understanding the dynamics of the archosaur radiation.

**Conclusions/Significance:**

Comprehensive numerical phylogenetic analyses demonstrate that both *Ctenosauriscus* and the Waldhaus taxon are members of a monophyletic grouping of poposauroid archosaurs, Ctenosauriscidae, characterised by greatly elongated neural spines in the posterior cervical to anterior caudal vertebrae. The earliest archosaurs, including *Ctenosauriscus*, appear in the body fossil record just prior to the Olenekian/Anisian boundary (c. 248 Ma), less than 5 million years after the Permian–Triassic mass extinction. These earliest archosaur assemblages are dominated by ctenosauriscids, which were broadly distributed across northern Pangea and which appear to have been the first global radiation of archosaurs.

## Introduction

Archosauria, consisting of the crown group of birds, crocodilians and their extinct relatives such as non-avian dinosaurs and pterosaurs [1], was the dominant terrestrial tetrapod clade for much of the Mesozoic, and continues to form an important component of extant ecosystems (>10,000 species). The clade is generally inferred to have originated in the Early Triassic (252.3–247.2 Ma: [2]) and the bird/crocodilian split has been proposed as a well-constrained calibration point for molecular clock estimates [3]–[6]. However, the earliest phase of archosaur history during the Early and early Middle Triassic is poorly understood, largely because of a paucity of fossils. As a result, relatively little is known about the timing, tempo, and major evolutionary patterns of the initial evolutionary radiation of archosaurs [6]–[11].

The first relatively well-known archosaur faunas in the fossil record are from the early Middle Triassic [3], [6], [7], [12]–[25]. Many of these assemblages, regrettably, suffer from poor chronostratigraphic control and their exact age is not well constrained. Although archosaurs surely originated and began to diversify during the Early Triassic, fossil material from this time interval is extremely scarce. Some of the oldest Early Triassic archosaur fossils are footprints, which unfortunately are abundant only locally, potentially controversial and difficult to interpret, and do not preserve much anatomical information (e.g. [9], [26], [27]). Early Triassic body fossils are, however, even rarer. Gower & Sennikov [7] discussed highly incomplete remains possibly pertaining to crown archosaurs from the latest Early Triassic Yarenskian Gorizont of Russia, and Nesbitt et al. [11] demonstrated that *Xilousuchus sapingensis* from the late Early Triassic to earliest Middle Triassic (see below) of China is a crown group archosaur (see also [6]). Documentation of additional material of Early Triassic and earliest Middle Triassic archosaurs is a crucial first step in establishing the pattern and process of the early archosaur radiation.

In light of the importance of early crown archosaur material, it is perhaps surprising that the historical taxon *Ctenosauriscus koeneni*, a sail-backed archosaur from the upper Middle Buntsandstein of Germany [28]–[30], has been almost entirely ignored by recent work on the early archosaur record (although see [18], [31]). When mentioned (largely in passing), it has generally been referred to as of Middle Triassic age [3], [22], stemming from the assertion of Krebs [30], [32] that the entire upper Middle Buntsandstein is of Anisian age. However, stratigraphic work supports a well-constrained latest Olenekian age for the part of the upper Middle Buntsandstein from which *Ctenosauriscus* was collected (see below), and thus *Ctenosauriscus* is one of the oldest known crown archosaur specimens, perhaps the oldest.

The aim of this contribution is to redescribe the holotype specimen of *Ctenosauriscus* as well as additional ctenosauriscid material from the earliest Middle Triassic of Germany, provide comparisons to other basal archosaurs, discuss the phylogenetic position of this material and the existence of a ctenosauriscid clade (a potential discrete group of sail-backed archosaurs that includes *Ctenosauriscus* and close relatives), and review the geographical and stratigraphic distribution of the earliest archosaurs.

### History of discovery

**Figure 1:**
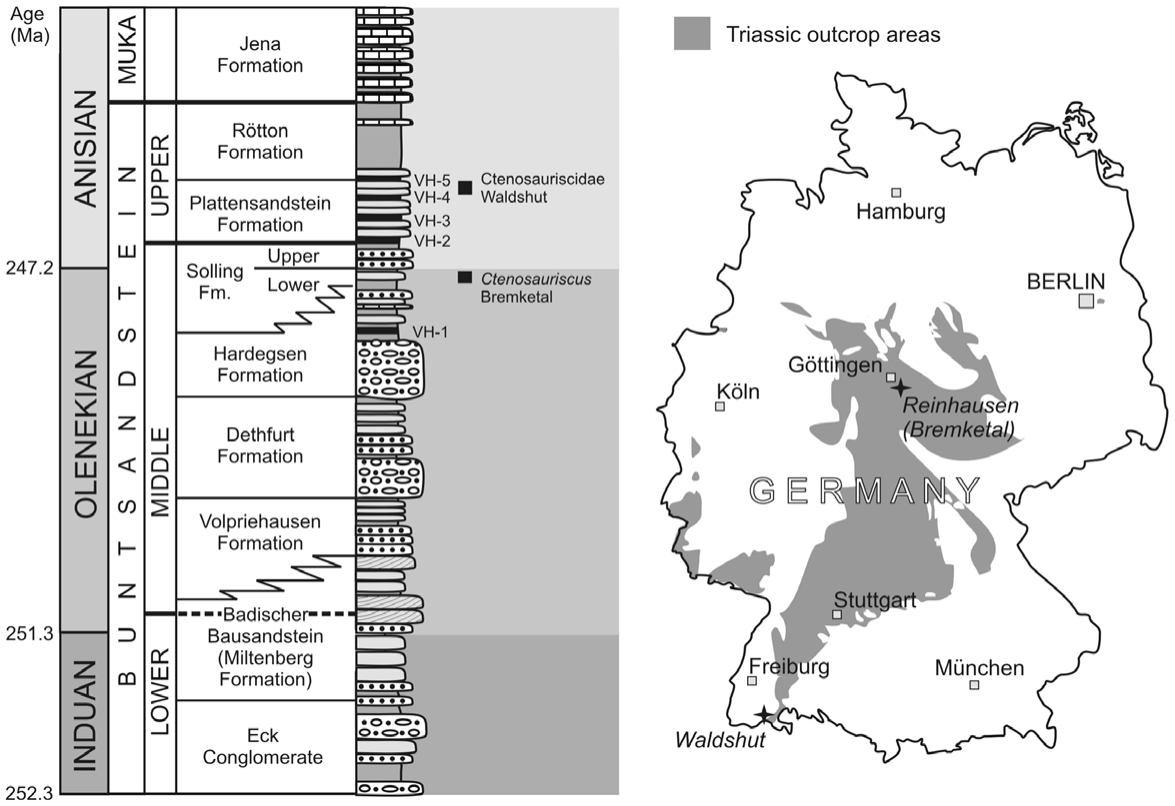
Stratigraphic and geographical data for German ctenosauriscid specimens. Stratigraphy of the German Buntsandstein (left), showing the stratigraphic levels at which *Ctenosauriscus* and the Waldshut ctenosauriscid were collected. Map of Germany (right) showing Triassic outcrops and the Bremketal and Waldshut localities.

**Figure 2:**
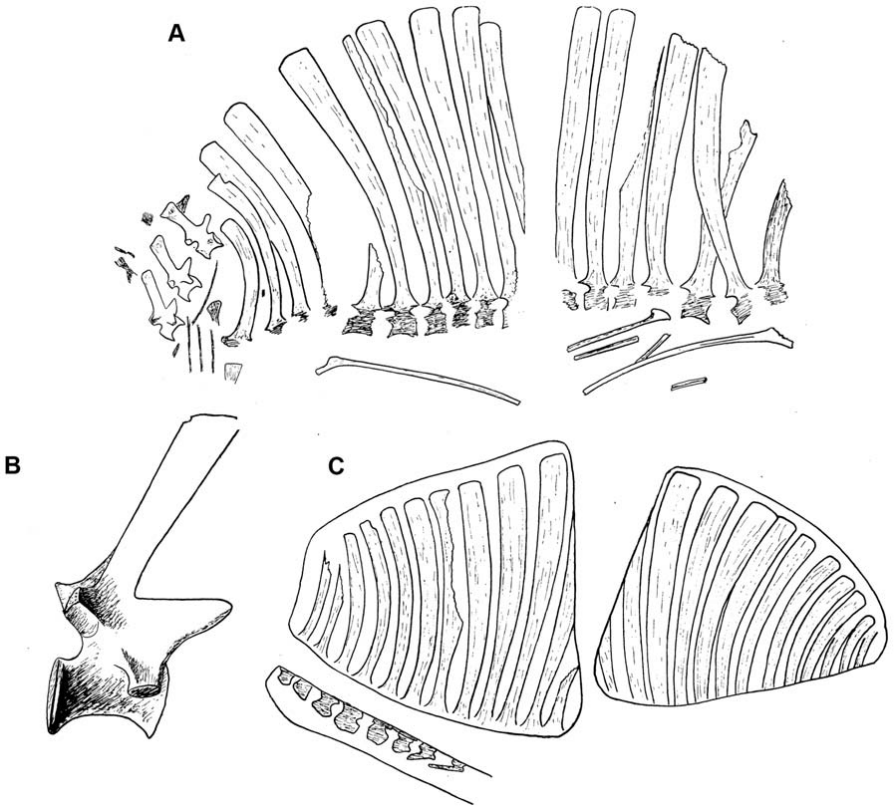
Historical depictions of the holotype specimen of *Ctenosauriscus koeneni*. A, slabs A1 (left) and A2 (right), from Huene (1914: fig. 1). B, cervical ‘3’, from Huene (1914: fig. 2). C, slabs B1 (right) and B2 (left) from Huene (1914: fig. 3). No scale bars were provided with any of these original illustrations.

The holotype specimen (GZG.V.4191) of *Ctenosauriscus koeneni* was discovered early in 1871 in a quarry at Bremketal ( =  “Bremke dell”) near Göttingen (Fig. 1), northern Germany, and later (November 1871) donated by master builder and architect Eduard Freise (1816–1885) to the University of Göttingen. The German palaeontologist Friedrich von Huene erected a new genus and species, *Ctenosaurus koeneni*, for the specimen in 1902 based upon examination of a photograph sent to him by Adolf von Koenen (1837–1915), professor of geology and palaeontology at the University of Göttingen. Huene [29] provided a more extensive description based upon direct examination of the specimen and additional preparation (Fig. 2A–C). Huene [29], [33] suggested that two individuals were represented by the holotype slabs (one individual represented by the slab and counterpart referred to below as slabs A1 and A2, and one represented by the slabs B1 and B2), and considered *Ctenosaurus* to represent a pelycosaurian-grade synapsid on the basis of its elongate neural spines. Abel [34] questioned the pelycosaurian affinities and considered *C. koeneni* to represent a temnospondyl similar to the sail-backed *Platyhystrix* (cf. [35]). Because the genus name *Ctenosaurus* was preoccupied, Kuhn [36] erected the replacement name *Ctenosauriscus*.

Krebs [30] noted that preparation undertaken by O. Abel between 1936 and 1938 had demonstrated that only a single individual is present, with its vertebral column split in half along a near sagittal plane. The specimen was redescribed and reinterpreted by Krebs [30], who recognised its archosaurian nature, referred it to Pseudosuchia (currently considered to represent the crocodilian total group), and noted similarities with a then undescribed sail-backed archosaur from the Middle Triassic (late Anisian) Lifua Member of the Manda Beds of Tanzania, more recently named *Hypselorhachis mirabilis* [22], [37]. Both *Ctenosauriscus* and *Hypselorhachis* were referred to the family Ctenosauriscidae by Krebs [30].

Zhang [15] described a new sail-backed pseudosuchian archosaur, *Lotosaurus adentus*, from the Middle Triassic (Anisian) Xinlingzhen Formation (Badong Group) of Hunan Province, China, and noted similarities with *Ctenosauriscus* and *Hypselorhachis*. Nesbitt [3], [18] recognised that the long mysterious archosaur taxon *Arizonasaurus babbitti* Welles, 1947 [38], from the early Anisian Holbrook Member of the Moenkopi Formation of Arizona (USA), is a sail-backed poposauroid pseudosuchian highly similar to *Ctenosauriscus*, and postulated the existence of a ctenosauriscid clade (a subgroup of poposauroids) including *Arizonasaurus*, *Ctenosauriscus*, *Lotosaurus*, *Bromsgroveia walkeri* from the Anisian Bromsgrove Sandstone Formation of England, and *Hypselorhachis*. Using a large numerical phylogenetic analysis, Nesbitt et al. [11] and Nesbitt [6] later documented a sister taxon relationship between *Arizonasaurus* and *Xilousuchus* (*Ctenosauriscus* was not included because its character scores overlapped completely with those of *Arizonasaurus*) within Poposauroidea, but found that these taxa did not group with *Lotosaurus*, therefore concluding that Ctenosauriscidae (*Arizonasaurus* + *Xilousuchus*) is a less inclusive clade than previously proposed (i.e., the ctenosauriscid clade postulated by Nesbitt [3], [18] is paraphyletic). Brusatte et al. [39] did not recover a monophyletic Ctenosauriscidae in their large numerical phylogenetic analysis of basal archosaurs, finding little resolution of relationships within the poposauroid clade.

### Institutional abbreviations

GZG, Geowissenschaftliches Zentrum der Universität Göttingen, Göttingen, Germany; IVPP, Institute of Vertebrate Paleontology and Paleoanthropology, Beijing, China; MSM, Arizona Museum of Natural History (formerly Mesa Southwest Museum), Mesa, Arizona, USA; NHMUK, Natural History Museum, London, UK; SMNS, Staatliches Museum für Naturkunde, Stuttgart, Baden-Württemberg, Germany; WARMS, Warwickshire Museum, Warwick, UK.

## Results

### Geographic and stratigraphic provenance

The holotype of *Ctenosauriscus koeneni* was collected from the “Solling-Bausandstein” (upper Middle Buntsandstein: Solling Formation) at Bremke dell in Niedersachsen (Lower Saxony), northern Germany (Fig. 1). Former quarries within this “Solling-Bausandstein” (“Solling building sandstone”) cropped out between Reinhausen and Bremke (western flank of the Eichsfeld–Altmark Swell), southeast of the city of Göttingen, with a maximum thickness of 40–45 m [40], [41].

The lower part of the Solling Formation (Wilhelmshausen Beds, Trendelburg Beds and Karlshafen Beds; cf. [42]) consists of coarse fluvial deposits that are dated on the basis of palynomorphs, conchostracans and palaeomagnetic data as latest Olenekian (late Spathian) [43]–[48]. These lower parts of the Solling Formation are separated by a short unconformity from the uppermost part of the formation, the Stammen Beds (equivalent to the Chirotherien-Sandstein of Thuringia, which yields a well-known vertebrate footprint assemblage), which is dated as earliest Anisian (Aegean) in age, also on the basis of palynomorphs, concostracans and palaeomagnetic data [43]–[47]. Although the Solling Formation was long considered entirely Lower Triassic in age, placement of the Olenekian/Anisian boundary within the uppermost Solling (approximately at the unconformity between the Karlshafen and Stammen beds) is now broadly supported [43]–[47].

The “Solling-Bausandstein” (e.g. [40], [49], [50]) of the northeastern Eichsfeld area can be correlated with the Trendelburg and Karlshafen Beds of the nearby Reinhardswald Trough (northern Hesse). These units (lower to middle parts of the Solling Formation) represent an environment dominated by a meandering to braided river system with a north trending direction, which deposited various clastic sediments, including sandstones, siltstones and claystones [41], [51]. Compositionally, the “Solling-Bausandstein” is a subarkose [52] and is predominantly greyish coloured in lower levels (as in the case of the sediment in which the holotype of *Ctenosauriscus koeneni* is preserved) with more reddish coloration in higher levels. In addition to *Ctenosauriscus*, invertebrates (insects, limulids) and predominantly plants have also been found in clay lenses within the “Solling-Bausandstein” of the Bremke dell area [53]–[55].

Based upon radioisotopic dates for the Olenekian–Anisian boundary (247.2 Ma [2]) and data on the number of short eccentricity Milankovitch cycles present within the Solling Formation, Kozur & Bachmann [43], [45] inferred a date of ∼247.5 Ma for the base of the Solling. This would suggest an age of approximately 247.5–247.2 Ma (latest Olenekian) for the holotype of *Ctenosauriscus koeneni*.

Additional ctenosauriscid material described here was collected by Franz Falkenstein between 1989 and 1991 from a temporary pit created during the building of an extension to the Waldhaus brewery, Waldshut district, Baden-Württemberg, southwest Germany (Fig. 1), and subsequently donated to the SMNS [31]. The pit exposed strata of the Upper Buntsandstein (Röt Formation), which is securely dated as of earliest Anisian (Aegean–early Bithynian) age due to the interfingering of marine and terrestrial sediments within this formation and the resultant possibility of biostratigraphic correlations using shallow marine invertebrates such as ammonites (e.g. [43], [46], [56]). Palaeomagnetic data also support an earliest Anisian age for the Röt Formation [43]. The ctenosauriscid material from Waldhaus stems from a massive greenish coarse sandstone (Rötquarzit) below the Violet Horizon 5 [57]; the latter is a paleosol within the Röt Formation from which most other Röt Formation vertebrates in southwestern Germany were collected [58]. The fauna comprises selachians, capitosaurian temnospondyls, and small protorosaurs (aff. *Amotosaurus rotfeldensis*), all of which occur as isolated bones. Single beds within the sandstone include swim tracks of tetrapods.

The ctenosauriscid material from Waldhaus is likely, therefore, to be of a broadly similar age to, or marginally older age than, *Arizonasaurus* from the Holbrook Member of the Moenkopi Formation, Arizona [18], [56], and is undoubtedly slightly younger than the holotype of *Ctenosauriscus koeneni*. However, the age difference between the Solling and Röt formations is minor, with no major unconformity separating the two and the top of the Röt estimated at ∼246 Ma [45], and so the holotype of *Ctenosauriscus koeneni* and the Waldhaus ctenosauriscid are likely separated from one another by at most 1.5 million years.

Huene [59] reported fragmentary tetrapod material, including vertebrae, from the uppermost Middle Buntsandstein of Nagold, near Calw, Baden-Württemberg, and Huene ([33]:259; [60]) mentioned that this collection included vertebrae with elongate neural spines, and suggested the presence of a taxon similar to *Ctenosauriscus*. The whereabouts of this material is unfortunately currently uncertain; it was formerly in the private collection of a Mr Bergrat Schüz [59], [60].

### Systematic Palaeontology

Archosauria Cope, 1869 [61] sensu Gauthier 1986 [1]

#### Phylogenetic definition

The least inclusive clade containing *Crocodylus niloticus* (Laurenti, 1768) [62] and *Passer domesticus* (Linnaeus, 1758) [63]. Definition follows Sereno [64], [65].

Pseudosuchia Zittel, 1887–1890 [66]

#### Phylogenetic definition

The most inclusive clade containing *Crocodylus niloticus* (Laurenti, 1768) [62] but not *Passer domesticus* (Linnaeus, 1758) [63]. Definition follows Nesbitt [6].

Poposauroidea Nopcsa, 1928 [67] sensu Weinbaum & Hungerbühler 2007 [68]

#### Phylogenetic definition

The most inclusive clade containing *Poposaurus gracilis* Mehl, 1915 [69], but not *Postosuchus kirkpatricki* Chatterjee, 1985 [70], *Crocodylus niloticus* (Laurenti, 1768) [62], *Ornithosuchus longidens* (Huxley, 1877) [71], or *Aetosaurus ferratus* Fraas, 1877 [72]. Definition follows Nesbitt [6].

Ctenosauriscidae Kuhn, 1964

#### Phylogenetic definition

The most inclusive clade containing *Ctenosauriscus koeneni* (Huene, 1902) [28] but not *Poposaurus gracilis* Mehl, 1915 [69], *Effigia okeeffeae* Nesbitt & Norell, 2006 [73], *Postosuchus kirkpatricki* Chatterjee, 1985 [70], *Crocodylus niloticus* (Laurenti, 1768) [62], *Ornithosuchus longidens* (Huxley, 1877) [71], or *Aetosaurus ferratus* Fraas, 1877 [72] (new definition).

#### Taxonomic content

*Ctenosauriscus koeneni* (v. Huene, 1902) [28], *Arizonasaurus babbitti* Welles, 1947 [38], *Xilousuchus sapingensis* Wu, 1981 [74], *Hypselorhachis mirabilis* Butler et al. 2009 [22], the “Waldhaus ctenosauriscid”, and possibly *Bromsgroveia walkeri* Galton, 1985 [75].

#### Diagnosis

The following characters support the monophyly of Ctenosauriscidae: neural spines of dorsal vertebrae greatly elongated (more than seven times taller than centrum height; more than four times height of neural spines of cervical vertebrae); neural spines of dorsal vertebrae are strongly curved in lateral view. A number of additional characters may also support the clade (see Discussion).

*Ctenosauriscus* Kuhn, 1964 [36]

*Ctenosauriscus koeneni* (Huene, 1902) [28]

“Saurierreste”; Ebert 1894: 11 [76]

“*Ctenosaurus Koeneni*, n. gen. n. sp.”; Huene 1902: 37–38, fig. 41 [28]

“*Ctenosaurus Koeneni* v. Huene”; Case 1907: 57, fig. 17 [77]

“*Ctenosaurus* v. Huene”; Zittel 1911: 195 [78]

“*Ctenosaurus Koeneni*”; Huene 1914: 496–498, fig. 1–2 [29]

“*Ctenosaurus* v. Huene”; Zittel 1923: 235 [79]

“*Ctenosaurus* F. von Huene”; Zittel 1927: 252 [80]

“*Ctenosaurus Koeneni* v. Huene”; Schmidt 1928: 386–387, fig. 1084 [81]

“*Ctenosaurus koeneni* Huene”; Huene 1932: 224 [60]

“*Ctenosaurus Koeneni* v. Huene”; Schmidt 1938: 120 [82]

“*Ctenosaurus koeneni*”; Abel 1939: 162, unnumb. fig. (135) [34]

“*Ctenosaurus koeneni*”; Kumm 1941: 21 [50]

“*Ctenosaurus koeneni*” Huene; Huene 1940: 286 [83]

“*Ctenosaurus koeneni*”; Huene 1942: 220–221, fig. 2 [35]

“*Ctenosaurus koeneni* Huene”; Huene 1956: 258, fig. 311 [33]

“*Ctenosaurus koeneni* Huene”; V'ûškov 1964: 241, fig. 191 [84]

“*Ctenosauriscus* n. n., für *Ctenosaurus* Huene 1902, präokk.”; Kuhn 1964: 324 [36]

“*Ctenosauriscus* Kuhn 1963 (*Ctenosaurus* Huene 1901, präokk.)”; Kuhn 1966: 122 [85]

“*Ctenosaurus koeneni* v. Huene”; Müller 1968: 486, fig. 577 [86]

“*Ctenosaurus koeneni* v. Huene”; Nagel & Wunderlich 1968: 15 [87]

“*Ctenosauriscus* (*Ctenosaurus* präokk.) *koeneni* (Huene 1902)”; Kuhn 1968: 15, fig. 3.2 [88]

“*Ctenosauriscus* [pro *Ctenosaurus* praeocc.] *koeneni* (v. Huene)”; Krebs 1969: 697–702, figs. 1–2, pl. 1–2 [30]

“*Ctenosauriscus* Kuhn 1961 (*Ctenosaurus* Huene 1902 präokk.)”; Kuhn 1971: 13, 38–39, fig. 12 [89]

“*Ctenosauriscus koeneni*”; Zhang 1975: 146 [15]

“*Ctenosauriscus koeneni*”; Krebs 1976: 91 [32]

“*Ctenosauriscus koeneni*”; Mader 1982: 318 [90]

“*Ctenosauriscus koeneni*”; Mader 1984: 138 [91]

“*Ctenosauriscus koeneni* (v. Huene)”; Müller 1985: 496, fig. 600 [92]

“*Ctenosauriscus*”; Carroll 1988: 619 [93]

“*Ctenosauriscus*”; Milner et al. 1990: 885 [94]

“*Ctenosauriscus koeneni* (Huene, 1902)”; Benton 1994: 392 [95]

“*Ctenosauriscus koeneni* (v. Huene)”; Ebel et al. 1998: 1 [31]

“*Ctenosauriscus koeneni*”; Nesbitt 2003: S236 [3]

“*Ctenosauriscus koeneni*”; Nesbitt 2005a: table 1 [18]

“*Ctenosauriscus koeneni* (Huene, 1902)”; Butler et al. 2009: 1023 [22]

“*Ctenosauriscus*”; Brusatte et al. 2010: 8 [39]

**Table 1:** Measurements of *Ctenosauriscus* (GZG.V.4191).

	CL	CHA	CHP	CPR	TH	SPH	SPB	SPA
Cervical ‘1’	50	35	35	56	128	72	19	36
Cervical ‘2’	50	e34	36	62	138	76	17	30
Cervical ‘3’	48	e32	37	57	143	86	16	
Dorsal ‘1’						206+	21	29
Dorsal ‘2’						260	21	40
Dorsal ‘3’						324	22	45
Dorsal ‘4’				56	439	383	e25	49
Dorsal ‘5’	e45	e33	33	56	496	440	27	52
Dorsal ‘6’	43	37	37	59	537	478	22	50
Dorsal ‘7’	48	30	34	46	547+	501+	29	e51
Dorsal ‘8’	45	32	35	50	588	538	29	46
Dorsal ‘9’	44	34	31	43	593	550	27	51
Dorsal ‘10’						557	24	48
Dorsal ‘11’						e546		
Dorsal ‘12’								42
Dorsal ‘13’							25	
Dorsal ‘14’						310+	18	
Sacral 1						472	21	42
Sacral 2						448	22	43
Sacral 3						419	29	37
Caudal 1						402	27	33
Caudal 2						358	25	32
Caudal 3						329	27	29
Caudal 4						293	22	27
Caudal 5						253	21	23
Caudal 6						211	19	
Caudal 7						178	17	19
Caudal 8						145	17	
Caudal 9						110+	15	

All measurements are in millimetres; blank entries indicate that the measurement is not preserved in the element or inapplicable. ‘+’ at the end of a measurement indicates that the measurement is a minimum estimate (e.g. because an element is incomplete). *Abbreviations*: *CHA*, centrum, dorsoventral height at anterior end; *CHP*, centrum, dorsoventral height at posterior end; *CL*, centrum, anteroposterior length; *CPR*, dorsoventral height from the base of the centrum to the dorsal margin of the prezygapophyses; *SPA*, anteroposterior length of apex of spine; *SPB*, anteroposterior length of base of spine; *SPH*, dorsoventral height of spine from dorsal margin of the prezygapophyses to spine apex; *TH*, total dorsoventral height of vertebra.

#### Holotype

**Figure 3:**
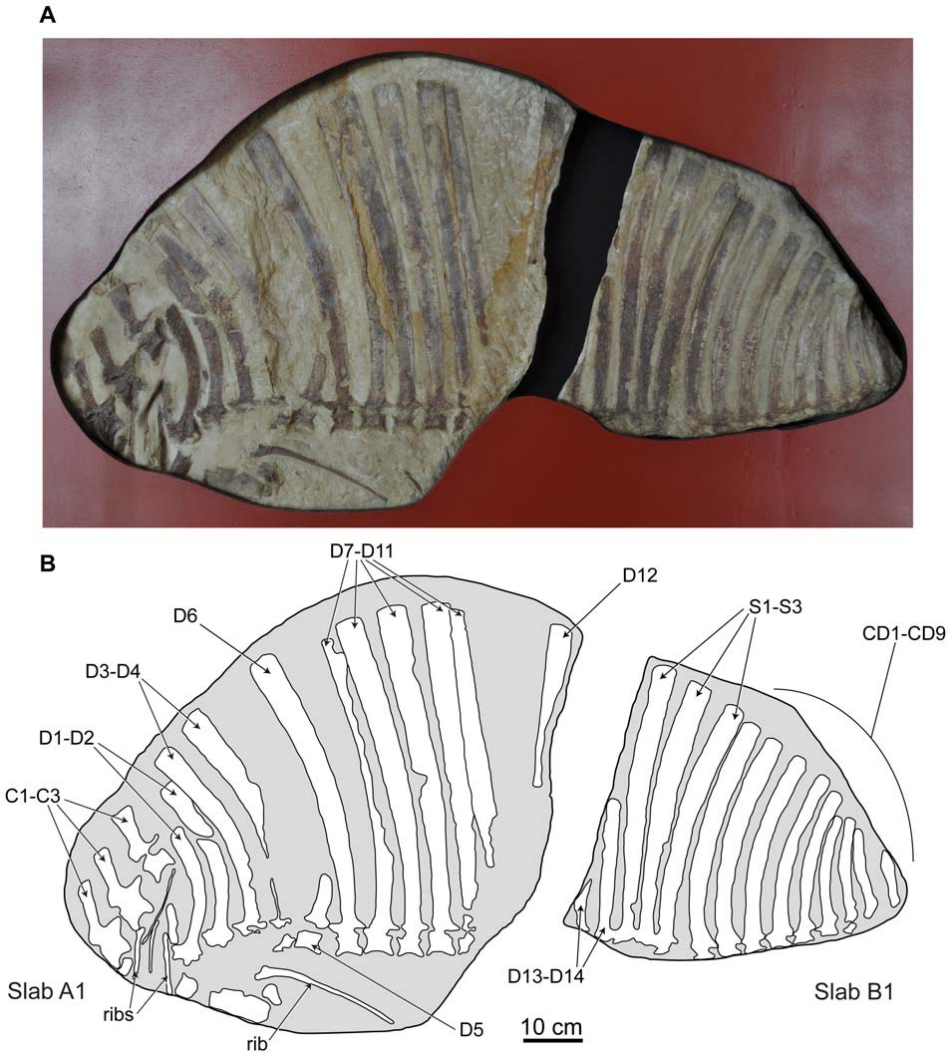
Holotype specimen of *Ctenosauriscus koeneni* (GZG.V.4191a–b). Photograph (A) and interpretative drawing (B) of slabs A1 and B1, forming together the part. *Abbreviations*: *C*, cervical vertebra; *CD*, caudal vertebra; *D*, dorsal vertebra; *S*, sacral vertebra.

**Figure 4:**
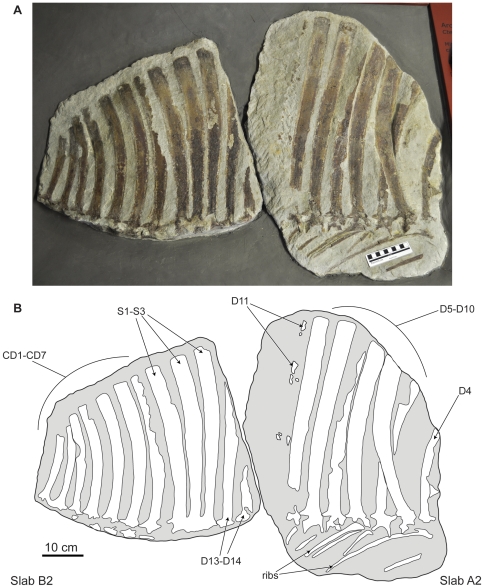
Holotype specimen of *Ctenosauriscus koeneni* (GZG.V.4191c–d). Photograph (A) and interpretative drawing (B) of slabs A2 and B2, forming together the counterpart. *Abbreviations*: *CD*, caudal vertebra; *D*, dorsal vertebra; *S*, sacral vertebra.

**Figure 5:**
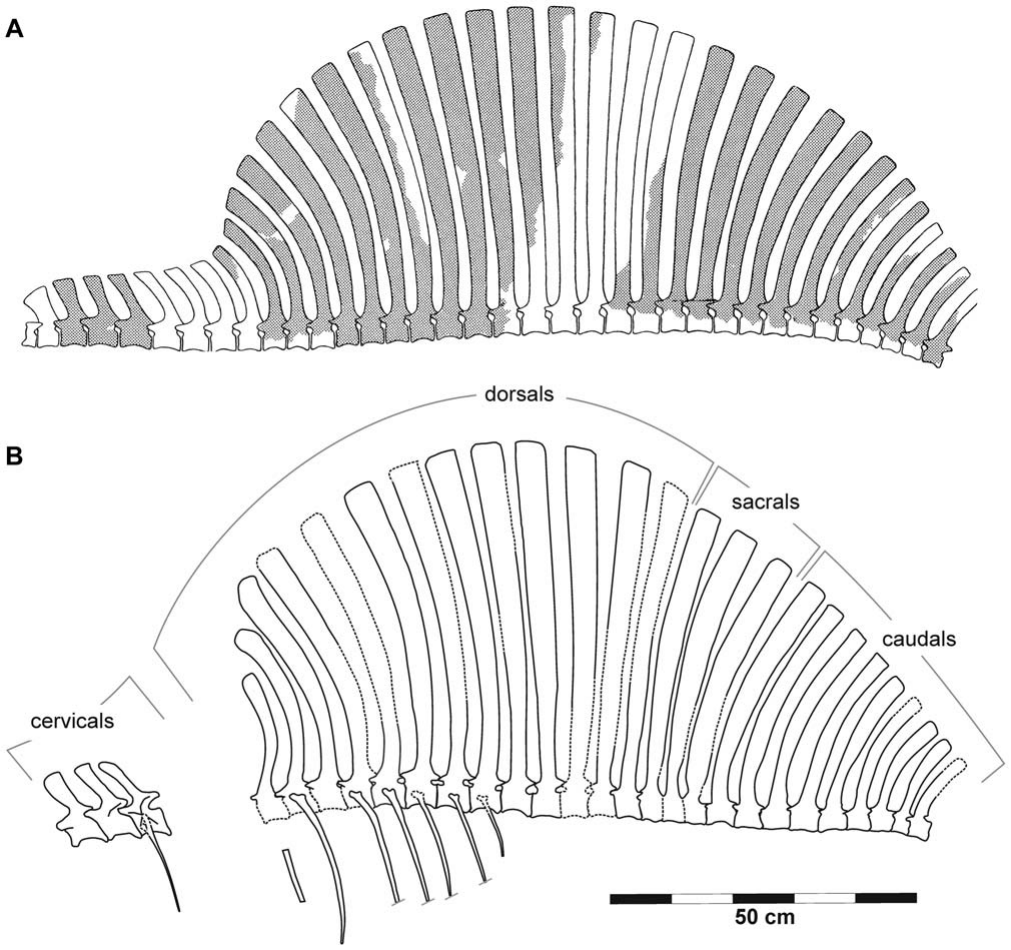
Reconstruction of the sail of *Ctenosauriscus*. (A) Reconstruction of Krebs ([30]:fig. 2). (B) New reconstruction, prepared by JJH.

**Figure 6:**
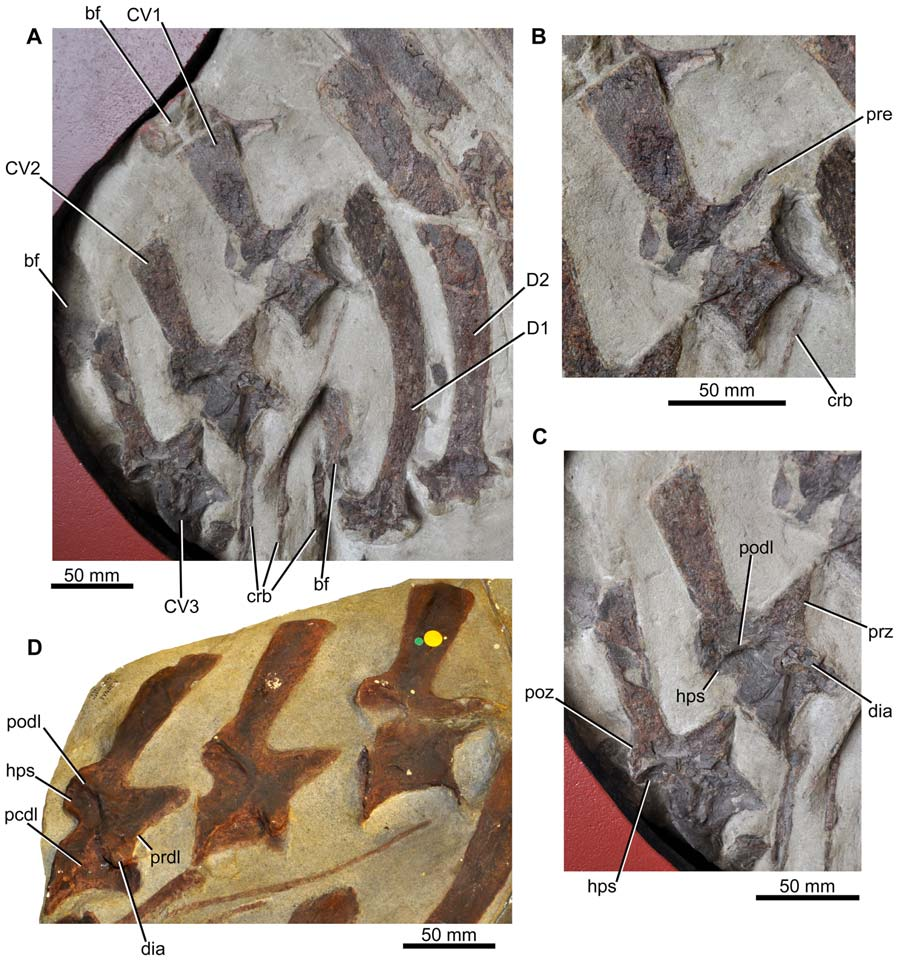
Cervical vertebrae of *Ctenosauriscus*. (A) Cervical vertebrae ‘1’–‘3’ of Slab A1 of GZG.V.4191 in right lateral view, with associated ribs and anterior dorsal vertebrae. (B) Close-up of cervical ‘1’ of GZG.V.4191. (C) Close-up of cervicals ‘2’ and ‘3’ of GZG.V.4191. (D) Cast of the cervical vertebrae (NHMUK R4976). *Abbreviations*: *bf*, bone fragment; *crb*, cervical rib; *CV*, cervical vertebra; *dia*, diapophysis; *D*, dorsal vertebra; *hps*, hyposphene; *pcdl*, posterior centrodiapophyseal lamina; *podl*, postzygodiapophyseal lamina; *poz*, postzygapophysis; *prdl*, prezygodiapophyseal lamina; *pre*, prezygapophysis.

**Figure 7:**
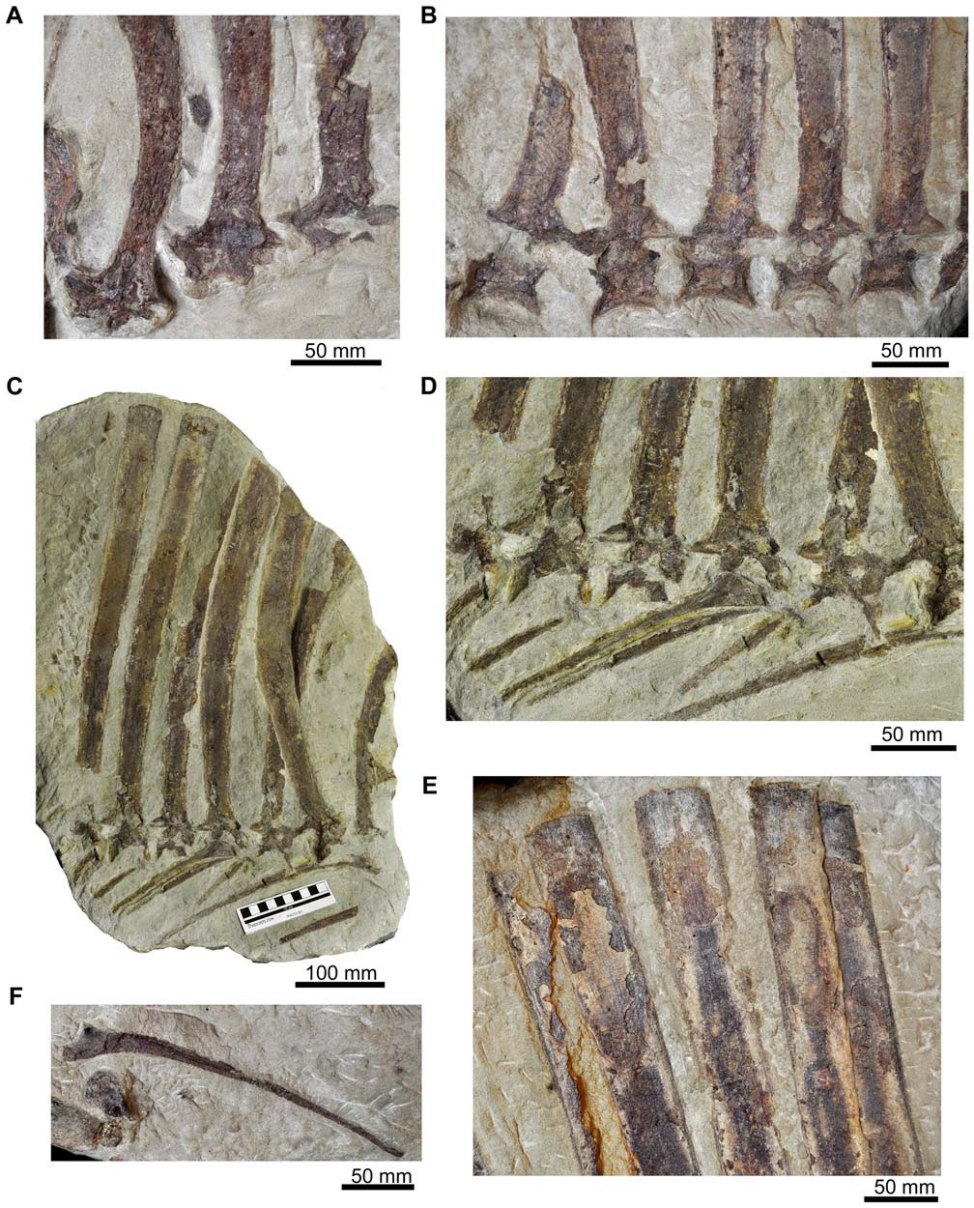
Dorsal vertebrae of *Ctenosauriscus* (GZG.V.4191). (A) Neural arches and neural spine bases of dorsal vertebrae ‘1’ to ‘3’ of Slab A1 in left lateral view. (B) Centra, neural arches and neural spine bases of dorsal vertebrae ‘5’ to ‘9’ of Slab A1 in left lateral view. (C) Slab A2 showing dorsals ‘4’ to ‘11’ and associated ribs in right lateral view. (D) Close-up of centra, neural arches, bases of neural spines and associated dorsal ribs of dorsals ‘5’ to ‘10’ of Slab A2 in left lateral view. (E) Apices of the neural spines of dorsal vertebrae ‘7’ to ‘11’ of Slab A1 in left lateral view; (F) Dorsal rib of Slab A1.

**Figure 8:**
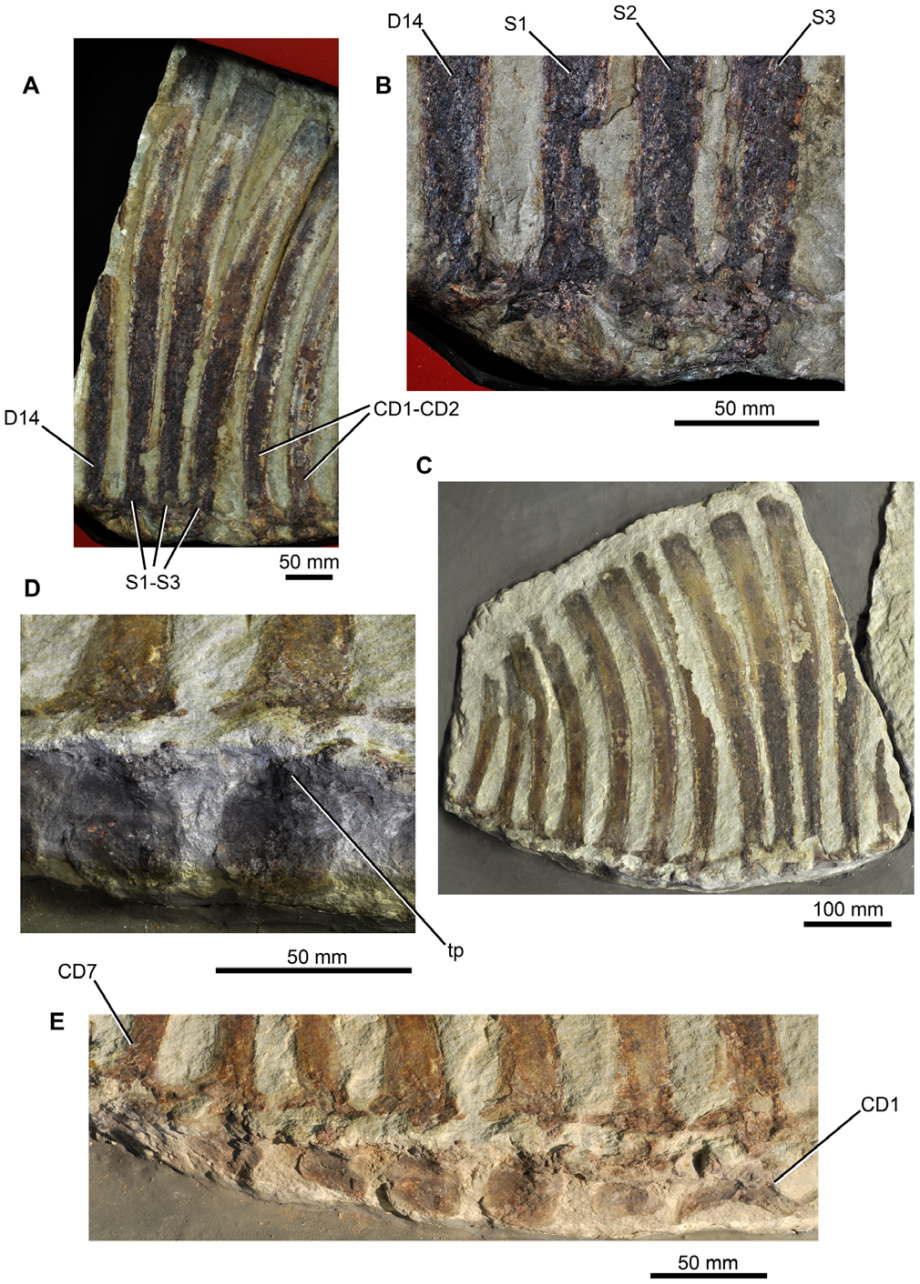
Sacral and caudal vertebrae of *Ctenosauriscus* (GZG.V.4191). (A) Sacral vertebrae of Slab B1 in left lateral view. (B) Close-up of the neural arches and neural spine bases of the sacral vertebrae of Slab B1 in left lateral view. (C) Slab B2 showing dorsals ‘13’ to ‘14’, sacrals 1–3, and caudals 1–7. (D) Lateral surfaces of the centra of caudals 3 and 4. (E) Lateral surfaces of the centra of caudals 1–7. *Abbreviations*: *CD*, caudal vertebra; *D*, dorsal vertebra; *S*, sacral vertebra; *tp*, transverse proces.

GZG.V.4191, partial vertebral column including parts of three cervical vertebrae, at least 13 or 14 dorsal vertebrae, three sacral vertebrae, nine anterior caudal vertebrae, five partial cervical ribs, eight partial dorsal ribs, unidentified bone fragments that may represent part of the pectoral girdle. Preserved on four sandstone blocks that together comprise the part and counterpart (Figs. 2, 3, 4, 5, 6, 7, 8).

The part is preserved in two pieces referred to here as slabs A1 and B1 (Figs. 2A–C, 3, 6, 7A, B, E, F, 8A, B; Huene [29]: Figs. 1A, 2, 3B; Huene [33]: fig. 311; Krebs [30]: fig. 1, pl. 1). These slabs (GZG.V.4191a–b) are currently in the museum of the GZG and permanently embedded within a wall display in a manner that makes examination of the edges of the blocks difficult (see Fig. 3). The counterpart is preserved in two pieces referred to here as slabs A2 and B2 (GZG.V.4191c–d; Figs 2A, C, 4, 7C, D, 8C–E; Huene [29]: Figs. 1B, 3A, C; Huene [33]: fig. 311; Krebs [30]: pl. 2) which are currently embedded in plaster within a display (see Figure 4). The anterior slabs (A1 and A2) and the posterior slabs (B1 and B2) do not fit together, and it is unclear by what distance they were originally separated from one another (see below).

A cast of slab A1 is available in the collections of NHMUK (NHMUK R4976) and was prepared by R. Jonas of Göttingen in 1923 (Fig. 6D). This slab preserves information on the cervical vertebrae that matches drawings provided by Huene [29], and suggests that the cervical vertebrae of GZG.V.4191 have been damaged subsequent to its initial discovery.

#### Type horizon and locality

“Solling-Bausandstein” (“Solling building sandstone”), upper Middle Buntsandstein (“Bunter”), Solling Formation (equivalent to the Trendelburg/Karlshafen Beds; latest Lower Triassic: Spathian, latest Olenekian).

Bremke dell ( =  “Bremketal”; probably from one of the former sandstone quarries “Immen-Berg” and “Grosser Hau”), east of the village of Reinhausen, approximately 10 km southeast of the city of Göttingen, Göttingen district, Niedersachsen (Lower Saxony), northern Germany (approximate coordinates: 51°27’54.44’’ N, 10°00’39.84’’ E). The type locality has been entered into the *Paleobiology Database* and is collection number 109489.

#### Diagnosis

Poposauroid archosaur characterised by the following unique combination of characters: (1) posterior cervical, dorsal, sacral and anterior caudal vertebrae with elongated neural spines that form a symmetrical ‘sail’ (shared with *Arizonasaurus*, *Xilousuchus*, *Lotosaurus* and *Hypselorhachis*); (2) extreme elongation of dorsal neural spines, with the longest neural spine more than 12 times the length of the centrum of its vertebra (probably shared with *Arizonasaurus*); (3) neural spines strongly expanded anteroposteriorly at their apices, reaching ∼190% of the anteroposterior length of their bases (probably shared with *Hypselorhachis*); (4) pre- and postzygapophyses of the dorsal vertebrae are large, robust processes that extend a substantial distance anterior and posterior to the articular faces of the centrum (probably shared with *Hypselorhachis*).

#### Comments

As discussed below, the incomplete nature of the holotype of *Ctenosauriscus koeneni* and of overlapping parts of the holotype skeletons of closely related taxa (*Arizonasaurus babbitti*, *Xilousuchus sapingensis*, *Hypselorhachis mirabilis*) limits comparisons, and we have been unable to identify unambiguous autapomorphies for *Ctenosauriscus*. However, the holotype can be distinguished from all other basal archosaurs by a unique combination of characters, and this allows us to provisionally retain *Ctenosauriscus* as a distinct taxon, pending discovery of more complete material.

### *Ctenosauriscus* - Description

Measurements of the specimen were previously provided by Krebs [30]; we provide a new and comprehensive set of measurements here (Table 1). Poor preservation means that it is not possible to determine the presence or absence of neurocentral sutures in any of the vertebrae; as a result, the ontogenetic status of the specimen is unknown. Limb bones, such as femora, are not preserved, and the preservation of vertebral material is poor. As a result, histological study to establish ontogenetic status is not feasible.

Slab A1 (GZG.V.4191a; NHMUK R4976; Fig. 3) is the largest and contains the anterior part of the vertebral column, including parts of three mid-to-posterior cervical vertebrae and at least 12 dorsal vertebrae (only the anterior 11 are present in the cast, NHMUK R4976). The best preserved vertebrae are the three cervicals (numbered as cervicals ‘1’–‘3’, beginning with the element that was positioned most anteriorly in life) that are exposed in right lateral view. The dorsal vertebrae (numbered here from the most anterior preserved dorsal vertebra, and thus referred to as dorsals ‘1’–‘12’) form a continuous series in close association, with some in near articulation (dorsals ‘6’–‘10’). The dorsals are oriented on the slab in the opposite direction to the cervicals and are therefore visible in left lateral view. Fragments of several ribs are present, as well as other, mostly unidentifiable, bone fragments (one of which was identified by Krebs [30] as a neural spine fragment, for a count of 13 dorsal vertebrae on this slab) concentrated around the cervicodorsal transition: some of these may represent additional vertebrae or parts of the pectoral girdle but preservation is too poor to be certain in most cases, and in any case they provide no useful anatomical details. Preservation is generally relatively poor, with many elements split sagittally between part and counterpart. Slab A2 (GZG.V.4191c) includes right lateral exposures of dorsals ‘4’–‘11’ and seven partial dorsal ribs (Fig. 4), but poor preservation means that no anatomical information is provided for the dorsal vertebrae beyond that which can be obtained from slab A1.

Slab B1 contains 14 partial vertebrae (GZG.V.4191b), including the two most posterior dorsals, three sacrals, and the nine most anterior caudals (Fig. 3). The relationship of slabs A1 and B1 to one another is unclear. Originally the slabs were reconstructed such that the last dorsal neural spine of slab A1 was connected to the first partial dorsal vertebra of slab B1 by plaster (see Krebs [30]: pl. 1). By contrast, Krebs [30] suggested that there was no overlap between the vertebral elements preserved on the two slabs and reconstructed a dorsal vertebra between the last preserved dorsal of slab A1 (dorsal ‘12’) and the first dorsal (here referred to as dorsal ‘13’) of slab B1. However, it is equally possible that there is no gap in the column at this point, or even that dorsal ‘12’ of slab A1 (represented by the spine apex only) and dorsal ‘13’ of slab B1 (represented by the spine base only) could be part of the same element, as shown in the reconstruction presented here (Fig. 5). In most elements of slab B1 only the spine and a fragmentary and poorly preserved neural arch are present, with fragments of the most dorsal parts of the centra visible in some cases. Slab B2 (GZG.V.4191d) contains 12 partial vertebrae, matching B1 with the exception of caudal vertebrae 8 and 9, which are not preserved.

In general, as reconstructed by Krebs ([30]: fig. 2; Fig. 5A) and here (Fig. 5B), the neural spines form a symmetrical sail with the length of the spines increasing from the cervical vertebrae through to the mid-to-posterior dorsal region, and then decreasing through the most posterior dorsals, sacrals and anterior caudals.

#### Cervical vertebrae

Cervicals ‘1’–‘3’ (Fig. 6) are identified as from the mid-to-posterior (i.e., approximately the 6^th^–8^th^ cervical vertebrae) cervical region based upon comparison to *Arizonasaurus* (MSM P4590; [18]:fig. 16F,G), because the centra are not greatly elongated compared to their dorsoventral height, the posterior face of the centrum is not strongly offset relative to the anterior face, and the neural spines are similar in height to those of the posterior cervicals of *Arizonasaurus* (MSM P4590; [18]:fig. 21). This interpretation differs from that of Krebs [30], who identified cervical ‘1’ as the third cervical vertebra. The mid-to-posterior cervicals of *Ctenosauriscus* possess centra that have lengths approximately 1.5 times their height in lateral view, with a ventral margin that is strongly arched dorsally, as in *Arizonasaurus* (MSM P4590).

The anterior and posterior faces of the centra are not exposed with the exception of the posterior face of cervical ‘3’, which is, however, not clearly visible due to the wall-mounting of the slab but which can be determined to be strongly concave (confirmed by examination of the cast, NHMUK R4976, Fig. 6D, in which the posterior surfaces of the centra of cervicals ‘1’ and ‘3’ are both strongly concave). The lateral surfaces of the centra are strongly concave anteroposteriorly, and the centra are therefore hourglass shaped in ventral view, as in *Arizonasaurus* (MSM P4590; [18]:Figs. 16,17). It is impossible to determine whether or not a ventral keel was present in *Ctenosauriscus* because the ventral margin of the centrum is not exposed. In cervicals ‘2’ and ‘3’ the broken base of the diapophysis is preserved anteriorly at the inferred level of the neurocentral suture (Fig. 6C: dia); the diapophyses of cervicals ‘2’ and ‘3’ were figured as more complete processes by Huene ([29]: Figs. 1a, 2), and are present in the cast (NHMUK R4976; Fig. 6D), and have thus presumably been damaged since his original description. A parapophysis cannot be recognised with certainty in any vertebra due to poor preservation. As in an anterior cervical of *Arizonasaurus* ([18]:fig. 17), a well-developed postzygodiapophyseal lamina (visible in cervicals ‘2’ and ‘3’; Fig. 6C: podl) arches anteroventrally from the lateral surface of the postzygapophysis. This lamina forms the lateral margin of the articular face of the postzygapophysis, and forms the anterodorsal margin of a fossa (the postzygapophyseal centrodiapophyseal fossa [96]) that covers the posterolateral surface of the neural arch (also present in *Arizonasaurus*, MSM P4590, [18]:fig. 17A, a.prz). A prezygodiapophyseal lamina occurs in cervical ‘3’ (based upon the cast, NHMUK R4976; Fig. 6D: prdl), and is uncertain in the other cervicals due to preservation. Posterior centrodiapophyseal laminae occur on cervicals ‘2’ and ‘3’, based upon the cast (NHMUK R4976; Fig. 6D: pcdl) and suggested by the drawing of cervical ‘3’ of Huene ([29]: fig. 2), but are poorly preserved in GZG.V.4191 due to damage to the lateral surfaces of the vertebrae.

Like the centra, the neural arches and spines of the cervicals are only exposed in lateral view, and are partially damaged, limiting the amount of information that can be obtained. The prezygapophyses are large triangular processes that extend a substantial distance beyond the anterior face of the centrum, similar to those of *Arizonasaurus* (MSM P4590; [18]:fig. 17A). The articular faces of the prezygapophyses are not exposed. The laterally flaring postzygapophyses are proportionally smaller than the prezygapophyses, and terminate posteriorly approximately level with the posterior face of the centrum. A ventral extending lamina beneath the postzygapophyses represents the hyposphene (cf. [11]:fig. 6C, D, hps). There is no epipophysis on the relatively well-preserved left postzygapophysis of cervical ‘3’. Between the postzygapophyses of cervical ‘3’, in posterior view, there is a small postspinal or spinopostzygapophyseal fossa [96], as also occurs in *Arizonasaurus* (MSM P4590; [18]:fig. 17C, p).

The neural spines increase slightly in height posteriorly from cervicals ‘1’ to ‘3’, with the spine of cervical ‘3’ being 120% of the height of the spine of cervical ‘1’ (Table 1). The spines are strongly compressed transversely and have anteroposteriorly short bases that are less than 40% of the total length of the centrum. The spines lack spine tables and osteoderms are not preserved and were likely absent. This is true of the entire vertebral column, because there are no signs of any bone fragments above the neural spines that could represent osteoderms. The spines expand in anteroposterior length towards their apex: this apical expansion is most marked in cervical ‘1’ (expansion of spine apex is 190% of that of the base) and becomes slightly less well developed more posteriorly (expansion of spine apex is 175% of the base in cervical ‘2’; the equivalent ratio for cervical ‘3’ is unknown). The neural spines are anterodorsally directed in lateral view, and the more posterior preserved cervicals have spines that are slightly more anteriorly directed. The apical margin of the spine is straight in lateral view. The posterior cervical neural spine figured by Nesbitt ([18]:fig. 21A; MSM P4590) for *Arizonasaurus* is similar to the neural spines of cervicals ‘2’ and ‘3’ of *Ctenosauriscus*.

#### Cervical ribs

At least three partial cervical ribs are positioned adjacent to the cervicals on slab A1 (Fig. 6A). The most anteriorly placed is an elongate and slender rod of bone; the more posterior ribs are thicker and more robust. The capitulum and tuberculum are not preserved in any of the exposed elements. The slender anterior rib is similar to those of the possible basal poposauroid *Qianosuchus* ([21]:fig. 3A) and of most ornithodirans, but differs from the shorter, stouter cervical ribs of *Lotosaurus* (IVPP V4913, 4880, 49271; [15]) and other pseudosuchians (e.g., *Postosuchus* [97]; *Ticinosuchus* [14]).

#### Dorsal vertebrae

The remaining 12 partial vertebrae on slab A1 are all from the dorsal column (Fig. 7A, B, E). The first of these, dorsal ‘1’, is likely to be from close to the beginning of the dorsal column (assuming a complete dorsal count of 15). Krebs ([30]:fig. 2) reconstructed four vertebrae (three entirely missing and a fourth represented by a neural spine fragment that we have been unable to identify with certainty: see above) between cervicals ‘1’–‘3’ and dorsal ‘1’. However, because the preserved cervicals appear to be from the mid-to-posterior cervical column (see above), the number of missing vertebrae may be fewer (perhaps as few as two, or more likely three) and neural spine height may have increased rapidly close to the cervicodorsal transition (see Fig. 5).

The centra of dorsals ‘1’–‘3’ are missing, while the centra of dorsals ‘4’–‘5’ are poorly preserved on slab A1 (they are more completely preserved on the counterslab, A2), with dorsal vertebra ‘5’ having been displaced slightly out of alignment with the rest of the column. The neural spines of dorsals ‘6’–‘11’ are relatively complete, and dorsals ‘6’–‘10’ preserve partial centra and neural arches, although these are poorly preserved. The height of the neural spines generally increases posteriorly through the cervical and dorsal series, reaching a maximum in dorsals ‘9’–‘10’. In dorsal ‘9’ the spine is 550 mm in length, 12.5 times the maximum length of the centrum of the same element (Table 1). The neural spine of dorsal ‘4’ is strongly arched anteriorly in lateral view; posteriorly the spines become progressively less strongly arched anteriorly and the spines of dorsals ‘10’–‘12’ are nearly straight. The orientation of the spines of dorsals ‘13’ and ‘14’ cannot be adequately assessed because of their incompleteness, but they were probably gently arched posteriorly as in the adjacent sacral vertebrae. As in the cervical vertebrae, the dorsal neural spines are transversely compressed and are anteroposteriorly narrow at their base but significantly expanded towards the apex (Fig. 7E): for example, in dorsal ‘9’ the anteroposterior length of the apex of the spine is 190% of the length of the base of the spine. The spines lack spine tables, and osteoderms are not preserved and were probably absent (see above). The apical margin of the spine is straight in lateral view in dorsals ‘2’ and ‘3’ (the apex is broken in dorsal ‘1’ and its apical shape cannot be assessed), but is slightly convex in dorsals ‘4’, ‘6’ and ‘8’–‘10’ (Fig. 7E; it cannot be assessed in other elements). The spines are not sufficiently well preserved to assess the presence or absence of pre- or postspinal fossae or muscle attachment ridges.

Little of the morphology of the neural arch can be documented in the dorsal vertebrae (Fig. 7A–D). The prezygapophyses and postzygapophyses are large and triangular in lateral view and both extensively overhang the centrum to a similar degree. The prezygapophyses are sufficiently well preserved in dorsals ‘1’, ‘3’, and ‘6’ to indicate that a discrete dorsal projection (the ‘dorsal lappet’ of Butler et al. [22]) is absent. The prezygapophysis is a discrete structure that is offset at 90 degrees to the spine, whereas the postzygapophysis merges more gradually with the spine along a curve. The articular faces of the zygapophyses are not exposed. Poor preservation prevents an assessment of the presence or absence of neural arch laminae, fossae or hyposphene/hypantrum articulations, and the transverse processes, parapophyses and diapophyses are eroded or poorly preserved on all dorsals.

The dorsal centra are also poorly preserved (Fig. 7B–D), but are slightly longer than high with ventral margins that are strongly arched dorsally in lateral view and possess concave anterior and posterior articular surfaces (it is possible to deduce the concavity of these articular surfaces, even though not exposed, because the centra are broken through their midline). In all of these features, the centra closely resemble those of *Hypselorhachis* [22] and *Arizonasaurus* [18]. The dorsal centra of *Lotosaurus*, however, have less concave (often to the point of being straight) ventral margins (IVPP V4913, 4880, 49271; [15]).

#### Dorsal ribs

One well-preserved dorsal rib is present on slab A1 ventral to dorsals ‘3’–‘7’ (Fig. 7F), and pieces of seven dorsal ribs are preserved on slab A2 in near life position (Fig. 7C), ventral to dorsal vertebrae ‘4’–‘10’. The first (most anterior) partial rib of slab A2 is a small fragment, and likely does not correspond to the adjacent dorsal ‘4’. The second preserved rib is located immediately below dorsal ‘4’, in near articulation with it. The remaining five ribs are in close association, and sometimes in contact or articulation with, dorsals ‘5’–‘9’: for example, the fifth of the ribs appears to be in articulation with dorsal ‘7’. The ribs are all elongate and slender, and are curved along their lengths. The capitulum and tuberculum are similar in size (both exposed on the second and fifth ribs of slab A2 and the lone dorsal rib of slab A1), and are separated by a concave notch. Well-preserved areas on the proximal half of the shaft are excavated by a longitudinal groove. It is unclear how far distally this groove continued, because all of the ribs are eroded beyond their midpoint. Where well preserved, especially in the first preserved rib of slab A2, the groove is quite sharp and centred along the shaft.

#### Sacral vertebrae

The three sacral vertebrae (Fig. 8A, B) are identified as such because their neural spines are near parallel to one another at their bases and are closely bunched together with smaller gaps between them than between the sacrals and the preceding and following vertebrae (there are gaps of 8 mm between the neural spines of sacrals 1–3, whereas there is a gap of 18 mm between the spines of dorsal ‘14’ and sacral 1 and a gap of 20 mm between the spines of sacral 3 and caudal 1). This suggests, therefore, that the three vertebrae were closely linked into a functional unit, and their zygapophyses appear to be fused to one another as is diagnostic for poposauroids [39], [98]; this character also occurs in *Arizonasaurus* [18], *Bromsgroveia* [18], *Lotosaurus* (IVPP V4913, 4880, 49271), *Effigia* [98], *Poposaurus* [18], *Sillosuchus* [99], and *Shuvosaurus* [98], [100].

The neural spines of the sacral vertebrae are posteriorly arched along their length, and the length of the spines decreases posteriorly. As in the cervical and dorsal vertebrae, the sacral neural spines are narrow at their bases and expanded at their apices, and their apical margins are convex in lateral view. No details of the neural arches or centra of the sacral vertebrae are available.

#### Caudal vertebrae

The neural spines of caudals 1–9 (Figs. 3, 4, 8C) are all posteriorly arched along their length, and the spines decrease in apicobasal height posteriorly along the column. As in the cervical, dorsal and sacral vertebrae, the neural spines of the most anterior caudals are narrow at their bases and expanded at their apices; however, the degree of expansion decreases posteriorly through the caudal series and from caudal 3 onwards the expansion is essentially absent and the spines are nearly parallel-sided. The apical margins of the caudal spines are convex in lateral view. The degree of curvature of the spines increases posteriorly, with those of caudals 2–4 being particularly strongly, but gradually, curved. The neural spines of caudals 5–7 differ from those of preceding caudal vertebrae in that the curvature involves a subtle but more discrete kink approximately halfway up the spine on the anterior margin. The neural spines of caudals 8 and 9 are straighter in lateral view than preceding elements.

Few details of the morphology of the neural arches of the caudal vertebrae are available; however, the triangular zygapophyses of the caudals are generally similar to those of the dorsal vertebrae and overhang the anterior and posterior faces of the centra.

On the bottom edge of slab B2, the right lateral surfaces of the centra of the first seven caudal vertebrae are preserved (Fig. 8D, E; identified as transverse processes by Huene [29]), and have thus been rotated from their original position by 90 degrees. All of the caudal vertebrae appear to be broken in the same place at the base of the neural spines; the break is most clearly seen on caudal 4 where the broken neural spine and the rotated centrum are separated from one another by approximately 10 mm, but the broken surfaces appear to fit together. The centrum of caudal 1 is substantially more elongate than those of the subsequent caudals and has a strongly arched ventral margin. The succeeding caudal centra are subquadrate in lateral view, similar to one another in size, and seem to lack strongly arched ventral margins. Their lateral surfaces are smoothly concave anteroposteriorly, but are substantially less waisted than the cervical and dorsal vertebrae. The transverse processes are broken off, but their bases are preserved in some cases (positioned at the anterodorsal margin of the centrum) and appear to be large: in caudal 3 the broken base of the transverse process is 12 mm in anteroposterior length and 15 mm deep.

### *Ctenosauriscus* - comparisons

As discussed above, there are strong similarities in vertebral morphology between *Ctenosauriscus* and *Arizonasaurus babbitti* [18] (MSM P4590), but detailed comparisons are complicated by the fact that most of the neural spines in the material of *Arizonasaurus* are incomplete, having fractured into numerous pieces. The two taxa appear to share extremely elongate neural spines (reaching more than 12 times the length of the centra in *Ctenosauriscus*), although accurate comparisons are hampered by the lack of exact associations between centra and neural spines in *Arizonasaurus*. One feature that appears to distinguish the two taxa is the fact that in *Arizonasaurus* the anterior and posterior margins of the preserved dorsal neural spines are subparallel along their length ([18]:fig. 21), expanding only subtly towards their distal end. By contrast, in *Ctenosauriscus* the spines are proportionally narrower anteroposteriorly at their base than those of *Arizonasaurus*, and they therefore become more strongly expanded towards their apex (with the anteroposterior length of their apices being up to 190% of the length of the bases). In addition, in *Arizonasaurus* the pre- and postzygapophyses are relatively short processes that do not extend extensively beyond the anterior and posterior margins of the centrum ([18]:fig. 19), whereas the pre- and postzygapophyses are proportionally larger and extend far beyond the anterior and posterior margins of the centrum in *Ctenosauriscus*. Nesbitt [18] suggested that the two taxa could be distinguished by the fact that the cervical neural spines do not arch as strongly anteriorly in *Arizonasaurus*. However, a preserved neural spine from the posterior cervicals of *Arizonasaurus* ([18]:fig. 21A) is similar to those of cervicals ‘2’–‘3’ of *Ctenosauriscus* (in both there is a gentle anterior curvature), so this proposed difference appears invalid.

*Hypselorhachis mirabilis* is known from only a single anterior dorsal vertebra [22], but differs from *Ctenosauriscus* in possessing an autapomorphic ‘dorsal lappet’ (small lobe-like dorsally directed projection) on the dorsal margins of the prezygapophyses. As suggested by Butler et al. [22], the neural spines may have been shorter relative to the dorsoventral height of the centrum in *Hypselorhachis* than in *Ctenosauriscus*. In the remainder of its morphology the holotype specimen of *Hypselorhachis* is similar to the anterior dorsal vertebrae of *Ctenosauriscus*; in particular, the two taxa are similar in that both have neural spines that are anteroposteriorly expanded at their apices, and have elongate and robust prezygapophyses (the postzygapophyses of *Hypselorhachis* are not preserved).

Comparisons to *Xilousuchus sapingensis* are limited to the mid-to-posterior cervical vertebrae, because dorsal vertebrae are unknown for this species and only a single sacral vertebra is known, but does not preserve its neural spine [11]. In overall proportions and morphology, the posterior cervical vertebrae of *Ctenosauriscus* and *Xilousuchus* are similar. The absence of well-preserved centra in *Ctenosauriscus* limits comparisons with the centra of *Xilousuchus.* Both appear to have a ventrally elongated lamina, possibly a hyposphene, between the postzygapophyses. The anterior extension of the prezygapophyses beyond the anterior margin of the centrum is proportionally greater in *Ctenosauriscus* than in *Xilousuchus*. The prezygapophyses of *Ctenosauriscus* project directly anteriorly such that their dorsal margin is horizontal in lateral view, whereas in *Xilousuchus* the prezygapophyses project strongly anterodorsally. A similar increase in neural spine height posteriorly through the cervical vertebrae is present in *Ctenosauriscus* and *Xilousuchus*. The neural spines of *Ctenosauriscus* have straighter anterior and posterior edges than *Xilousuchus*; this difference results in a straight but anterodorsally inclined neural spine in *Ctenosauriscus*, whereas the neural spines of *Xilousuchus* arc anterodorsally along their lengths. As in *Ctenosauriscus*, the cervical neural spines of *Xilousuchus* are substantially expanded at their apices relative to their bases. The anterodorsal and posterodorsal corners of the apices of the neural spines of *Ctenosauriscus* (specifically, the first cervical preserved) are acute angles, whereas the anterodorsal and posterodorsal corners of the apices of the neural spines of *Xilousuchus* are more rounded.

The morphology of *Lotosaurus* has been described only briefly in the literature [15], but two of us (SLB, SJN) have personally examined many specimens, including the type material (IVPP V4913, 4880, 49271). We do not provide a detailed description of the vertebral morphology of *Lotosaurus* here, as this taxon is currently under study (J. Liu et al., pers. comm.). However, it is important to provide a general comparison with *Ctenosauriscus*. Although both *Ctenosauriscus* and *Lotosaurus* possess elongate neural spines, the anatomy of these spines (and the vertebral centra) differs in detail. The middle cervical neural spines of *Lotosaurus* are proportionally taller and slimmer anteroposteriorly than those of the corresponding vertebrae (cervicals ‘1’ and ‘2’) in *Ctenosauriscus*. Furthermore, the middle cervical neural spines of *Lotosaurus* are swollen distally into a small, laterally expanded, rounded ridge, whereas those of *Ctenosauriscus* are not, and do not expand appreciably toward their distal end, whereas those of *Ctenosauriscus* funnel out in anteroposterior length towards their apices. The morphology of the neural spines of the dorsal vertebrae is even more markedly different in the two taxa. Those of *Lotosaurus* are proportionally shorter dorsoventrally (compared to the height of the centrum) and in many cases (especially at the anterior and middle portions of the sail) are very strongly expanded anteroposteriorly at their distal apices, much more so than in *Ctenosauriscus*. As in the cervical spines, lateral expansions at the distal end of the neural spines are present in *Lotosaurus*, but absent in *Ctenosauriscus*. Furthermore, the anterior dorsal neural spines of *Lotosaurus* are less strongly curved than those of *Ctenosauriscus* [18]. Finally, the dorsal centra of *Lotosaurus* often have straight ventral margins in lateral view, contrasting with the strongly concave margins of *Ctenosauriscus*. One feature potentially shared by the two taxa is that the transverse processes of the anterior caudal vertebrae of *Lotosaurus* (IVPP V4913, 4880, 49271) are proportionally large and swollen, possibly similar to those inferred for *Ctenosauriscus*, although poor preservation and missing anterior caudals in other poposauroids renders broader comparisons difficult.

Comparisons to the putative ctenosauriscid *Bromsgroveia* are limited because the vertebral column of *Bromsgroveia* is poorly known, and no neural spines are known with certainty (one specimen that has been described as a possible ctenosauriscid neural spine from the Middle Triassic Otter Sandstone Formation of England does not possess any clear diagnostic features of vertebral spines and may represent a rib: [16], [18], [22], [24], [94]). *Bromsgroveia* resembles *Ctenosauriscus* in possessing the poposauroid character of at least three sacral vertebrae, the zygapophyses of which are fused to one another [16], [18], [101]. No differences between the two taxa can be identified at present.

Other poposauroids such as *Qianosuchus mixtus* [21], *Effigia okeeffeae* [73], *Poposaurus gracilis* [102], *Sillosuchus longicervix* [99], and other Triassic archosauriforms differ from *Ctenosauriscus* in lacking strongly elongated neural spines that form a symmetrical sail [6], [39]. The unusual trilophosaurid archosauromorph *Spinosuchus caseanus* from the Tecovas Formation (Late Triassic) of Texas (USA) possesses elongate neural spines in the dorsal, sacral, and anterior caudal series, but differs in that these spines are proportionally shorter, terminate apically in broad triangular expansions (in the dorsal series), and have thin sheet-like lateral expansions [103].

Ctenosauriscidae indet.

“Waldshuter Rauisuchier”; Ebel et al. 1998: 3 [31]

#### Material

**Figure 9:**
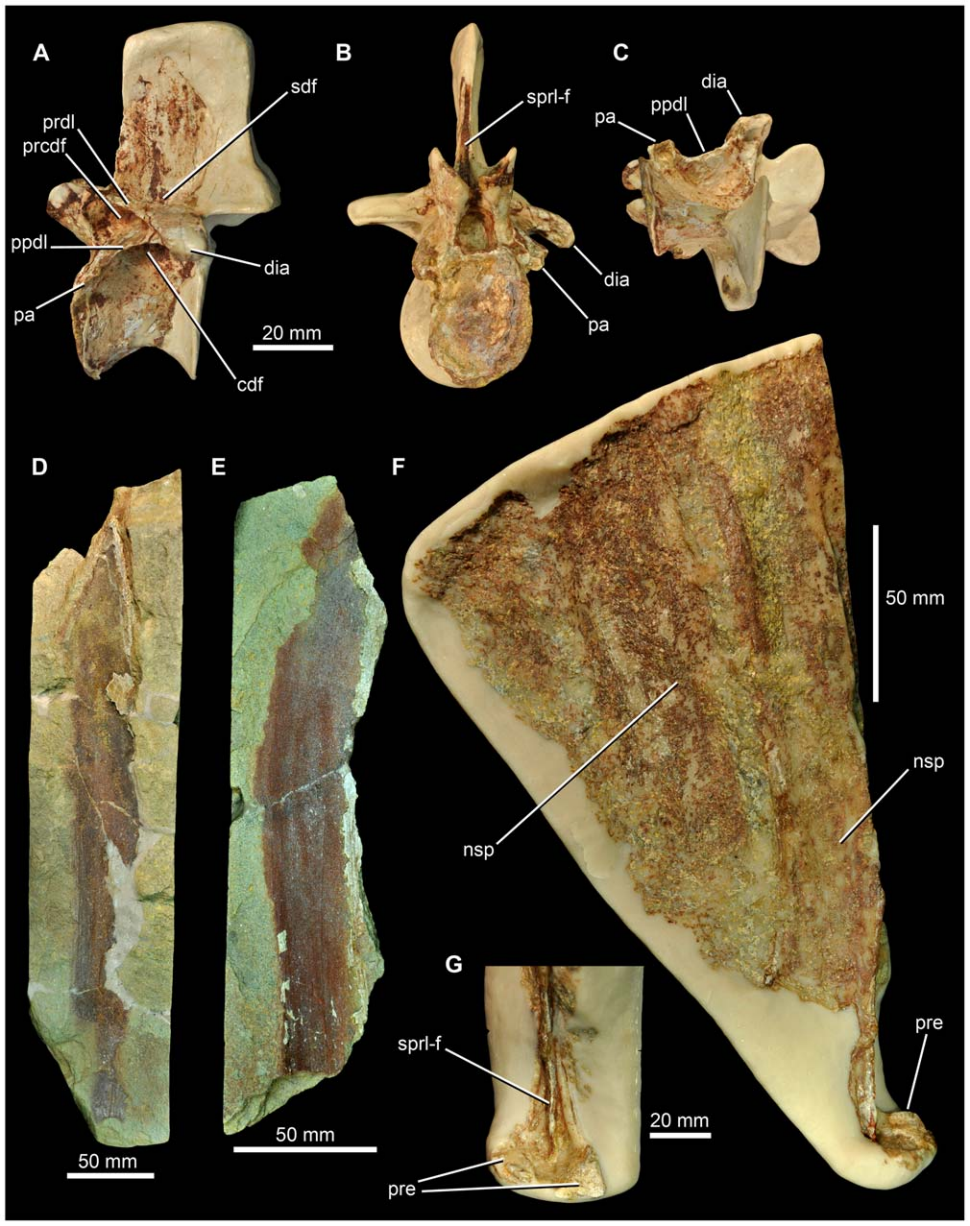
Archosaur material from the Waldhaus brewery, Waldshut district, southern Germany. SMNS 91402, anterior dorsal vertebra in lateral (A), anterior (B) and ventral (C) views. SMNS 91403 (D) and SMNS 91404 (E), impressions of elongate neural spines. SMNS 91405 (F), two elongate neural spines with a close-up of the prezygapophyses (G). Abbreviations: *cdf*, centrodiapophyseal fossa; *dia*, diapophysis; *nsp*, neural spine; *pa*, parapophysis; *ppdl*, paradiapophyseal lamina; *prcdf*, prezygapophyseal centrodiapophyseal fossa; *prdl*, prezygodiapophyseal lamina; *pre*, prezygapophysis; *sdf*, spinodiapophyseal fossa; *sprl-f*, spinoprezygapophyseal fossa.

**Figure 10:**
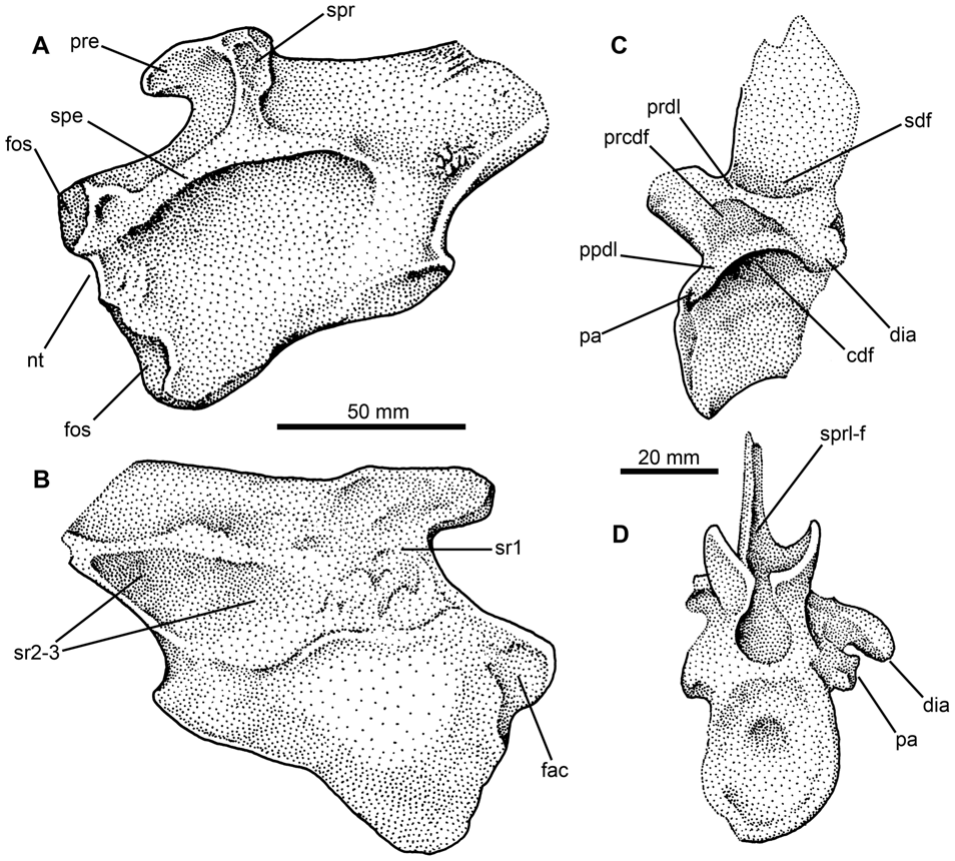
Archosaur material from the Waldhaus brewery, Waldshut district, southern Germany. SMNS 91401, left ilium, stippled drawings in lateral (A) and medial (B) views. SMNS 91402, anterior dorsal vertebra, stippled drawings in lateral (C) and anterior (D) views. Abbreviations: *cdf*, centrodiapophyseal fossa; *dia*, diapophysis; *fac*, facet on medial surface of pubic peduncle; *fos*, fossae on pubic peduncle; *nt*, notch in anterior margin of pubic peduncle; *pa*, parapophysis; *ppdl*, paradiapophyseal lamina; *prcdf*, prezygapophyseal centrodiapophyseal fossa; *prdl*, prezygodiapophyseal lamina; *pre*, preacetabular process; *sdf*, spinodiapophyseal fossa; *spe*, supraacetabular expansion or rim; *spr*, supraacetabular ridge; *sprl-f*, spinoprezygapophyseal fossa; *sr1*, *sr2*–*3*, sacral rib scars.

**Figure 11:**
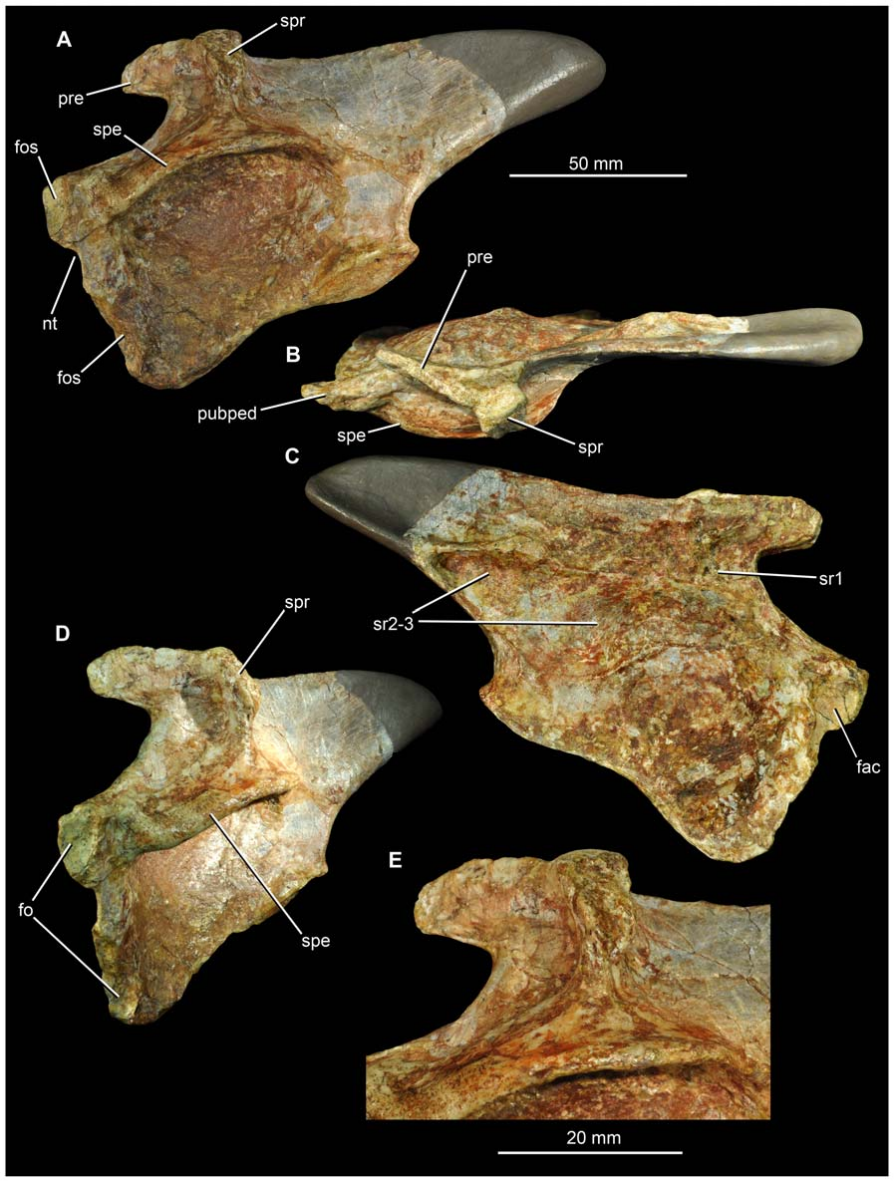
Archosaur ilium (SMNS 91401) from the Waldhaus brewery, Waldshut district, southern Germany. Left ilium in lateral (A), dorsal (B), medial (C), anterolateral (D) views, and close-up of the supraacetabular ridge (E). Abbreviations: *fac*, facet on medial surface of pubic peduncle; *fos*, fossae on pubic peduncle; *nt*, notch in anterior margin of pubic peduncle; *pre*, preacetabular process; *pubped*, pubic peduncle; *spe*, supraacetabular expansion or rim; *spr*, supraacetabular ridge; *sr1*, *sr2*–*3*, sacral rib scars.

SMNS 91402, partially preserved anterior dorsal vertebra (Figs. 9A–C, 10C, D). SMNS 91405, two elongate neural spines (Fig. 9F, G). SMNS 91403, 91404, partially preserved elongate neural spines (Fig. 9D, E). SMNS 91041, left ilium (Fig. 10A, B, 11).

#### Horizon and locality

Röt Formation, Upper Buntsandstein (earliest Middle Triassic: early Anisian: Aegean–Bithynian). Waldhaus brewery, Waldshut district, Baden-Württemberg, southwest Germany (47°40′58″ N, 08°09′26″ E). The locality has been entered into the *Paleobiology Database* and is collection number 109490.

### Waldhaus ctenosauriscid - description

It is unclear whether this material pertains to a single or multiple individuals of a single taxon or multiple taxa, as no clear associations exist. The size and morphology of the elements described here are consistent with the morphology present in ctenosauriscid pseudosuchians (e.g., *Arizonasaurus*, *Ctenosauriscus*), and thus we hypothesise that they belong to a single taxon. Additional fragmentary material (including vertebral and rib fragments and isolated carnivorous teeth) from this site may also belong to the same taxon, but are not described here due to their incompleteness.

#### SMNS 91402

This specimen is a partial dorsal vertebra (Figs. 9A–C, 10C, D), identified as from the anterior to mid-dorsal column based upon the position of the parapophysis, and consisting of a partial centrum (the posterior face of which is missing), partial neural arch (the postzygapophyses and left transverse process are missing), and the base of the neural spine. The missing parts of the vertebra have been reconstructed with white plaster, which covers original bone surface in some places. The vertebra has suffered some transverse distortion such that the right side is displaced posterior to the left side. Out of the remains found from Waldhaus, this specimen is the least diagnostic because it lacks any clear synapomorphies with ctenosauriscids or poposauroids.

The neurocentral suture is completely closed, indicating that this probably represents an osteologically mature individual [104]. The anterior face of the centrum has an oval outline that is taller (29.5 mm) than wide (23 mm), with a concave articular surface. The centrum is very strongly compressed transversely at its midpoint (7 mm wide at its narrowest point), although this may have been exaggerated to a small degree by post-mortem compression. Immediately ventral to the inferred position of the neurocentral suture is a shallow, elliptical, and blind fossa (likely non-pneumatic, as such shallow fossae on vertebral centra are widespread in archosauriforms: [105]), the long axis of which extends anteroposteriorly (Fig. 10C). The maximum preserved length of the centrum, from the anterior face to the base of the posterior centrodiapophyseal lamina, is 31 mm, suggesting a complete length of at least 35 mm. The ventral margin of the centrum is strongly arched dorsally in lateral view (i.e., is strongly concave).

The parapophysis is damaged and missing on the right side; on the left side it is placed at the most anterior margin of the vertebra, at the same level as the inferred position of the neurocentral suture (Figs. 9A, B, 10C, D: pa). The parapophysis is compressed in an anterodorsal–to–posteroventral direction, and has a concave, subcircular, ventrally facing articular facet. The diapophysis is not well-preserved on the left side (and is missing on the right side), but is positioned at the end of a short (approximately 25 mm long), slightly downturned, posterolaterally extending transverse process (Figs. 9A, B 10C, D: dia). A well-defined paradiapophyseal lamina extends between the parapophysis and the diapophysis (Figs. 9A, 10C: ppdl). A well-defined posterior centrodiapophyseal lamina is present, and the paradiapophyseal and posterior centrodiapophyseal laminae define the anterior and posterior margins of a very deep, dorsomedially extending, funnel-shaped centrodiapophyseal fossa [96] (Figs. 9A, 10C: cdf) which appears to be blind.

A well-defined prezygodiapophyseal lamina extends from the anterodorsal corner of the diapophysis to the prezygapophysis (Figs. 9A, 10C: prdl); the prezygodiapophyseal lamina and the paradiapophyseal lamina form the dorsal and posteroventral margins of a very deep, triangular, prezygapophyseal centrodiapophyseal fossa (Figs. 9A, 10C: prcdf; it is unclear whether or not this fossa is blind at its base). The anterior margin of this fossa is defined by a broadly rounded, buttress-like prezygoparapophyseal lamina. A postzygapophyseal centrodiapophyseal fossa [96] also occurs, but only its base is preserved.

The neural canal is only exposed anteriorly, and is oval in outline. Lateral to the neural canal, the lobe-like prezygapophyses extend anterodorsally beyond the anterior margin of the centrum. In anterior view, the prezygapophyses are very steeply inclined, facing dorsomedially at approximately 50 degrees to the horizontal. Their articular surfaces are strongly concave transversely. A narrow vertical slot between the prezygapophyses likely represents the hypantrum, as in other rauisuchians [6], [39].

Only the base of the neural spine is preserved, and so it is not possible to determine if the neural spine of this element was elongate. A narrow slot-like spinoprezygapophyseal fossa is present at the base of the neural spine anteriorly (Figs. 9B, 10D: sprl-f), and is bordered by spinoprezygapophyseal laminae. There are no ‘dorsal lappets’ at the points where the spinoprezygapophyseal and prezygodiapophyseal laminae meet, unlike the condition in *Hypselorhachis* [22]. An elliptical, anteroposteriorly extending, blind fossa (spinodiapophyseal fossa) is present on the dorsal surface of the base of transverse process (Figs. 9A, 10C: sdf), at the point where it merges with the base of the neural spine.

#### SMNS 91405

This specimen consists of at least two, and possibly three, partial neural spines within a block of sediment that has been embedded in plaster (Fig. 9F, G). One neural spine is moderately well exposed with paired prezygapophyses visible; the second is a very poorly exposed spine, and its margins are difficult to ascertain because of poor preservation and the close similarity in colour of the bone and sediment. The transversely compressed first spine has a maximum length of approximately 210 mm, although it is unclear whether it is complete at its apex. The spine is approximately 30 mm in anteroposterior length close to its base, and as far as can be determined maintains a near constant anteroposterior length along its length, i.e. it does not expand towards the apex. The spine curves gently along its length, away from the prezygapophyses, and this suggests that it is from the posterior part of the sail (i.e. from the posterior dorsal to anterior caudal region). The anterior edge of the spine is a sharp ridge that extends all the way to its base, where it bisects the midline of (and subdivides) a small, shallow, spinoprezygapophyseal fossa. On both sides of the fossa are the poorly preserved prezygapophyses, the articular surfaces of which face dorsomedially at around 30 degrees to the horizontal. The second spine is similar in its dimensions to the first, with approximately 114 mm being visible, and has an anteroposterior length of approximately 30 mm. It also curves posteriorly towards its apex. Parts of a third spine may be preserved in one corner of the block, but this cannot be determined with certainty.

#### SMNS 91403, 91404

SMNS 91403 is an impression of an elongate, transversely compressed bone, with only some small fragments of bone remaining (Fig. 9D). It almost certainly represents an impression of a ctenosauriscid neural spine, based on its transverse compression (the preserved bone fragments are ∼2 mm thick), great length (the preserved portion exceeds 325 mm), relatively constant anteroposterior length (∼35 mm), and very slight curvature along its length. A second impression, SMNS 91404 (Fig. 10E), has even fewer fragments of bone preserved, but is more strongly curved along its length. The length as preserved is 203 mm, and the anteroposterior length as preserved is ∼33–35 mm.

#### SMNS 91401

This element, a left ilium (Figs. 10A, B, 11), was previously described and figured by Ebel et al. [31]. The bone is relatively complete, but lacks the distal half of the postacetabular process (the missing part of the process was reconstructed with plaster, as shown by Ebel et al. [31]:fig. 1). The preserved portion of the element is 133 mm long (from the anterior tip of the pubic peduncle to the preserved distal margin of the postacetabular process), and we estimate the complete length as approximately 190 mm in length based upon comparisons to *Arizonasaurus*. Thus, this specimen is almost identical in length to the specimen of *Arizonasaurus* (MSM P4590) described by Nesbitt [18], which has a left ilium that is 195 mm long.

**Figure 12:**
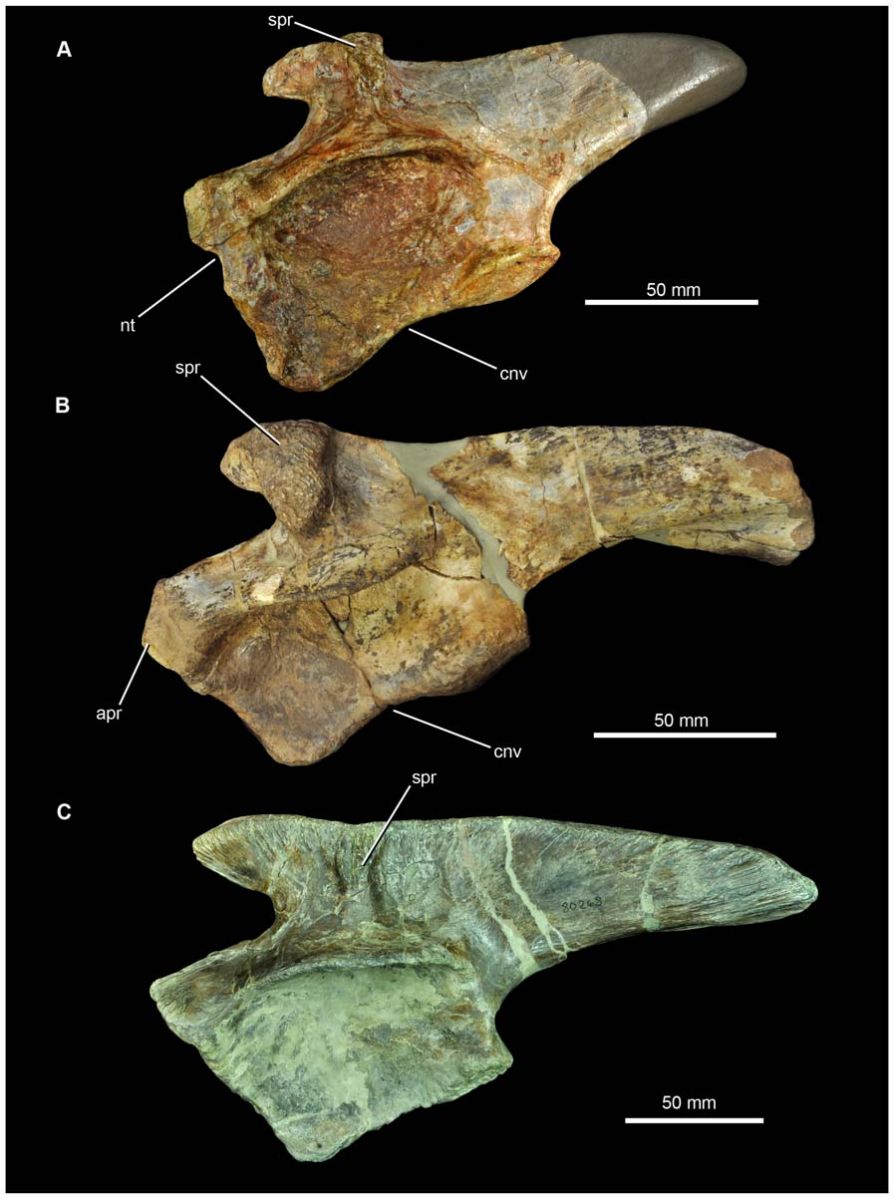
Left ilia of pseudosuchian archosaurs from the Middle Triassic in lateral view. (A) SMNS 91401, Waldhaus brewery, Waldshut district, southern Germany. (B) MSM 4590, referred specimen of *Arizonasaurus babbitti*, Holbrook Member of the Moenkopi Formation (Middle Triassic: early Anisian) of Arizona. (C) SMNS 80268, referred specimen of *Batrachotomus kupferzellensis*, Erfurt Formation (Middle Triassic: late Ladinian) of southern Germany. *Abbreviations*: cnv, concave margin of ischial peduncle; *nt*, notch in anterior margin of pubic peduncle; *spr*, supraacetabular ridge.

The preacetabular process is small and finger-like (Figs. 10A, 11A: pre), highly similar to that of *Arizonasaurus* (MSM P4590; Fig. 12) and *Bromsgroveia* (WARMS G.3, NHMUK R2549 [cast]; [16], [101]) ([39]: character 117). The “finger-like” condition refers to a preacetabular process that is much shallower than the postacetabular process, stops far short of the anterior margin of the ilium, and rises above the remainder of the ilium dorsally, as it is separated from the rest of the dorsal margin of the ilium by a subtle notch, best visible in medial view (Figs. 10B, 11C). Some larger rauisuchians, such as *Batrachotomus kupferzellensis* (SMNS 80268), also have somewhat of a “finger-like” precetabular process, but it is proportionally deeper and extends further anteriorly than in *Arizonasaurus*, *Bromsgroveia*, and SMNS 91401. In SMNS 91401, the preacetabular process projects anteromedially when seen in dorsal view.

A large, prominent, and rugose supraacetabular ridge or crest occurs above the acetabulum on the lateral surface of the blade (Figs. 10A, 11A: spr), as is characteristic of ‘rauisuchians’ (see discussion in Gower [106]), although this ridge is not as anteroposteriorly thick as in *Arizonasaurus* (MSM P4590) and is set further posteriorly than in either *Arizonasaurus* (MSM P4590) or *Bromsgroveia* [16], with the result that the preacetabular process of SMNS 91401 is longer anterior to the ridge than in *Arizonasaurus* (MSM P4590). The ridge is separated in SMNS 91401 from the supraacetabular expansion or rim (which forms the dorsal roof of the acetabulum: Figs. 10A, 11A, B, D, E: spe) by a gently dorsoventrally concave and smooth margin. The ridge rises subvertically at its base, but towards its apex it curves anteriorly and merges into the preacetabular process. The dorsal surface of the ridge is marked by a deep concavity (Fig. 11B), which is absent in *Arizonasaurus* (MSM P4590) and *Bromsgroveia* (WARMS G.3, NHMUK R2549 [cast]), the former of which has a convex dorsal surface marked by linear striations. The anterior surface of the ridge is excavated in SMNS 91401 (Figs. 10A, 11D), forming a small shallow fossa that faces mostly laterally but also anteriorly.

The supracetabular rim is prominent and strongly expanded, extending more than 25 mm lateral to the acetabular wall. The rim is laterally convex in dorsal view. Beneath the rim the acetabular wall is extensive, proportionally deeper than in *Arizonasaurus* (MSM P4590) or *Bromsgroveia* (NHMUK R2549 [cast]), and extends ventrally as a thin flange whose lateral surface is smoothly concave. The medial surface of the acetabular wall is convex at its centre but becomes flatter towards the ventral margins. The ventral margin is raised as a low rim. A similar rim is absent in *Arizonasaurus* (MSM P4590) but present in *Bromsgroveia* (NHMUK R2549 [cast]).

The pubic peduncle is transversely compressed, with a small fossa on its lateral surface anteriorly (Figs. 10A, 11A, D: fos). There is a small, discrete notch in the anterior margin of the peduncle (Figs. 10A, 11A: nt) that is absent in *Arizonasaurus* (MSM P4590), in which the anterior margin of the peduncle is broadly concave. This discrete notch is also absent in *Bromsgroveia*, in which the anterior margin of the pubic peduncle is flat to gently convex [16] (NHMUK R2549 [cast]). The medial surface of the peduncle adjacent to this notch is excavated by a concave facet (Figs. 10B, 11C: fac) with a smooth bone surface, the long axis of which extends dosoventrally. This facet is absent in *Arizonasaurus* (MSM P4590), *Bromsgroveia* (WARMS G.3, NHMUK R2549 [cast]), and *Batrachotomus* (SMNS 80206, 80272), and is likely a unique character of SMNS 91401. The function of this facet is uncertain. Its surface is smooth and appears to be articular, but it is positioned too far ventral to have articulated with a sacral rib (it is separated from the sacral rib scar by an 18 mm margin). Ventral to the discrete notch the anterior margin of the peduncle is nearly straight, and there is a small fossa that may mark the ventral termination of the pubic articulation (a similar fossa is also present in *Bromsgroveia*: NHMUK R2549 [cast]). If correctly identified, this implies that a contact between the pubic and ischium was absent in this specimen, as in *Arizonasaurus* [18].

The ischial peduncle is well preserved. The articular surface can be divided into two parts, as in *Arizonasaurus* (MSM P4590). Posteriorly, there is a raised ovoid facet that is twice as long as wide. The articular surface of this facet faces laterally and ventrally, is flat to slightly concave, and is surrounded by a slightly raised rim on all sides. A similar ovoid facet is present in *Arizonasaurus* (MSM P4590) but is not defined by a raised rim of bone. A stout and gently raised lamina links the posterolateral surface of the ovoid facet with the ventral surface of the postacetabular process. Anterior to the ovoid facet, the ischial peduncle is transversely thin and its margin is concave in lateral view. A similar morphology is present in *Arizonasaurus* (MSM P4590), in which this entire area forms a narrow contact with a convex region of the proximal ischium. The concave margin of the ischial peduncle is a poposauroid character, and has been interpreted as indicating a semi-perforate acetabulum (e.g. [39]), but this does not appear to necessarily be the case based upon *Arizonasaurus* in which the margin is concave but the acetabulum is not perforate [18].

On the medial surface of the ilium there is a continuous and marked scar for the sacral ribs, three of which can be inferred to have articulated here (Figs. 10B, 11C: sr1, sr2–3). The shapes and positions of the sacral rib scars are very similar to those of *Arizonasaurus* (MSM P4590) and *Bromsgroveia* as well as *Batrachotomus*. The first rib scar is positioned immediately posterior to the notch separating the preacetabular process from the pubic peduncle. This scar is C-shaped because it extends onto the bases of both the preacetabular process and the pubic peduncle. A subtle transverse ridge appears to separate the first scar from the second scar, which is the largest of the three. The third scar is smallest and is triangular, tapering in depth posteriorly. Its anterior margin is defined by a very subtle ridge that separates it from the second scar, but its dorsal and ventral margins are formed by prominent laminae. The dorsal and ventral laminae merge with one another posterior to the third sacral rib scar. This differs from the condition in *Arizonasaurus*, in which the laminae are separated by a groove or furrow that runs along the ventral surface of the postacetabular process.

### Waldhaus ctenosauriscid - comparisons

The ilium from Waldhaus (SMNS 91401) shows numerous differences from *Arizonasaurus* (MSM P4590), including: the vertically extending rugose ridge above the acetabulum is proportionally narrower anteroposteriorly, and is set further posteriorly; dorsal surface of the ridge is concave with a smooth surface texture; acetabulum is proportionally deeper; pubic peduncle is transversely compressed along its entire length with a small notch in the anterior margin; no distinct anterior process on the pubic peduncle (presence of this feature is a possible autapomorphy of *Arizonasaurus* [18]); absence of groove on ventral surface of postacetabular process separating ridges that define the scar for sacral rib 3 dorsally and ventrally (Fig. 12). Despite these differences, there are a number of similarities between SMNS 91401 and *Arizonasaurus*, including the finger-like preacetabular process, the gently concave margin of the ischiadic peduncle, and the inferred presence of three sacral vertebrae, with a similar pattern of sacral rib attachments. SMNS 91402 closely resembles the mid-dorsal vertebra of *Arizonasaurus* figured by Nesbitt ([18]: 19A, B; MSM P4590), sharing with it the strongly arched ventral margin of the centrum, a near identical arrangement of vertebral laminae and fossae, an oval neural canal, a downturned and posterolaterally directed transverse process, and compressed, ventrally facing parapophyses. Although poorly preserved, SMNS 91403–91405 are also similar to the neural spines of *Arizonasaurus*, in being highly elongate and curved along their length with apparently subparallel anterior and posterior margins.

Comparisons of the Waldhaus material to *Xilousuchus* are not possible because of the lack of overlapping bones. Comparisons to *Ctenosauriscus* are limited, because the ilium of the latter taxon is unknown and the dorsal vertebral centra are poorly preserved. As far as can be determined, the dorsal centrum and neural arch appear to be similar in SMNS 91402 and *Ctenosauriscus*, particularly in the strongly arched ventral margin of the centrum and the prominent, large prezygapophyses. The neural spines SMNS 91403–91405 may differ from *Ctenosauriscus* in having subparallel anterior and posterior margins, although they are insufficiently complete to confirm this distinction.

The holotype specimen of *Hypselorhachis mirabilis* is similar to SMNS 91402, sharing with it the strongly arched ventral margin of the centrum, a nearly identical arrangement of vertebral laminae and fossae, robust prezygapophyses, and a posterolaterally directed transverse process. However, the neural canal is broader than high (rather than higher than broad) in *Hypselorhachis*, the transverse process is not downturned, and the parapophysis faces laterally rather than ventrally [22]. Moreover, the single autapomorphy of *Hyselorhachis*, a ‘dorsal lappet’ at the point where the spinoprezygapophyseal and prezygodiapophyseal laminae meet, is absent in SMNS 91402. The neural spines SMNS 91403–91405 may differ from *Hypselorhachis* in having subparallel anterior and posterior margins, although this cannot be confirmed (see above).

SMNS 91401 differs from *Bromsgroveia* in a number of features: the vertically extending rugose ridge above the acetabulum is more vertically oriented and is set further posteriorly; dorsal surface of the ridge is concave; pubic peduncle is transversely compressed along its entire length with a small notch in the anterior margin [16], [101]. SMNS 91401 shares with *Bromsgroveia* the shallow, finger-like preacetabular process, the gently concave margin of the ischiadic peduncle, and the inferred presence of three sacral vertebrae, with a similar pattern of sacral rib attachments. The neural spines of *Bromsgroveia* are unknown; an anterior dorsal centrum of *Bromsgroveia* ([16]:fig. 4C, D) is considerably elongated relative to SMNS 91402 with a ventral margin that is only slightly arched dorsally and well-defined foramina within the neural arch fossae.

SMNS 91401 and the ilium of *Lotosaurus* are substantially different in many features. *Lotosaurus* lacks the “finger-like” morphology of the preacetabular process present in SMNS 91401 and other ctenosauriscids (*Arizonasaurus*, *Bromsgroveia*), and instead has a much deeper, but anteroposteriorly shorter, preacetabular process. Furthermore, *Lotosaurus* possesses a truly incipiently open acetabulum (and therefore a proportionally shallower acetabular surface than in SMNS 91401 and other ctenosauriscids), and possesses a rugose ridge above the acetabulum that is thicker and which curves more strongly anterodorsally than in SMNS 91401. Furthermore, although the postacetabular process of SMNS 91401 is broken, the preserved portions indicate that it was likely proportionally longer (anterposteriorly) but shallower (dorsoventrally) than in *Lotosaurus*, as the postacetablar process of *Lotosaurus* is remarkably short, deep, and almost square shaped. SMNS 91401 and *Lotosaurus* do share some features of the ilium, however, including a rugose ridge that extends anterodorsally onto the preacetabular process and which fans out both anteriorly and posteriorly at its dorsal apex.

SMNS 91401 differs from the ilium of *Poposaurus* primarily in lacking the elongate, downturned preacetabular process, in possessing a more vertically orientated supraacetabular ridge that does not overhang the acetabulum, and in lacking a truly incipiently open acetabulum [68], [102]. *Poposaurus* additionally lacks the elongated neural spines present in SMNS 91403–91405.

In summary, the Waldhaus material (assuming that it represents a single taxon) can be distinguished with confidence from the ctenosauriscids *Arizonasaurus*, *Hypselorhachis*, and *Bromsgroveia*, as well as the poposauroid *Lotosaurus*, whereas the elongate neural spines separate this material from all non-ctenosauriscid archosauriforms. The morphology of the neural spines may distinguish the Waldhaus specimens from *Ctenosauriscus*, from which it is also separated by a short stratigraphic distance, but this cannot be confirmed at present. The Waldhaus ctenosauriscid may represent a new taxon, but we prefer to leave this material unnamed pending recovery of more complete material from the Röt Formation.

### Phylogenetic analysis

In order to determine the phylogenetic position of *Ctenosauriscus* and the Waldhaus taxon within Archosauria and to test the monophyly of a clade of high-spined pseudosuchian archosaurs (Ctenosauriscidae), we performed two phylogenetic analyses using modified versions of the datasets presented by Brusatte et al. [39] and Nesbitt [6] (see Methods, below).

#### Brusatte et al. [39] reanalysis

First, before reporting the results of the modified Brusatte et al. [39], some comments on the original analysis are warranted. Brusatte et al. [39] analyzed their 55 taxon, 187 character matrix with a heuristic search (because the dataset is too large to examine all possible trees) implemented in an older version of PAUP [107]. Because several iterations of the analysis, including various subsets of taxa and characters and performed on different computer platforms, returned consistent results, the authors did not elect to use the parsimony ratchet [108] or other methods that rapidly explore a large number of tree islands. These methods are often useful when analyzing large datasets with a great deal of homoplasy (as is the case with basal archosaurs), as these datasets are often prone to get stuck on individual tree islands during heuristic searches. If this is the case, then tree space is not fully explored and many additional most parsimonious trees (MPTs), or shorter trees, may remain undiscovered.

**Figure 13:**
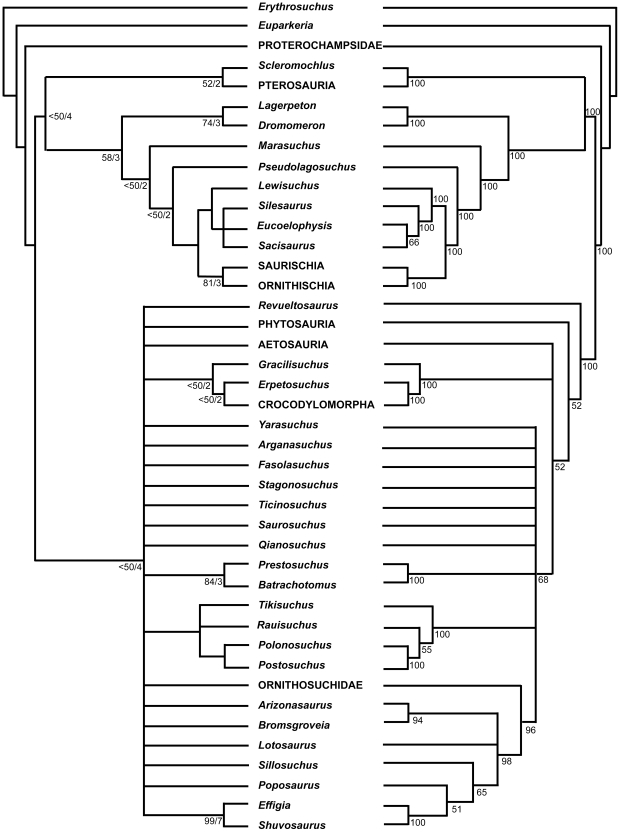
Strict consensus (left) and 50% majority rule consensus (right) resulting from reanalysis of the Brusatte et al. [**39**] dataset in TNT (see text for search parameters). On the strict consensus, numbers next to clades denote Bremer/bootstrap values, the latter calculated with 1000 replications. On the majority rule consensus, numbers next to clades denote the percentage of most parsimonious trees in which that clade is recovered. All clades without support values have a bootstrap percentage of less than 50% and a Bremer support of 1 (i.e., they fall apart in the strict consensus of all MPTs and trees one step longer than the most parsimonious trees).

In the course of the current study, we analyzed the original Brusatte et al. [39] dataset using TNT, a phylogenetic software package that implements the parsimony ratchet and other methods to more effectively explore tree space (multiple tree islands) [109]. As a first step, we analyzed the matrix under the “new technology search” option, using sectorial search, ratchet, tree drift, and tree fuse options with default parameters. We instructed the program to locate the minimum length tree in 10 replicates (which tries to sample as many tree islands as possible), and then analyzed these generated trees under traditional TBR branch swapping (which more fully explores each tree island). We found a much larger number of most parsimonious trees than reported by Brusatte et al. [39], as our search resulted in 3,324 MPTs (length = 741; consistency index = 0.302; retention index = 0.677). The strict consensus and majority rule consensus of these trees is presented here in Figure 13. In the strict consensus, the relationships within Avemetatarsalia remain identical to those reported by Brusatte et al. [39], but the relationships within Pseudosuchia ( =  Crurotarsi in the analysis of Brusatte et al. [39]) are substantially less resolved. Most importantly, a monophyletic Rauisuchia and a Crocodylomorpha + Aetosauria clade are not recovered, unlike in the original analysis. The majority rule consensus, however, does show a monophyletic Rauisuchia containing Ornithosuchidae.

We also note that França et al. [110] recently reanalyzed the Brusatte et al. [39] dataset using slightly different search parameters in TNT and recovered a similar strict consensus, although it differs in some small details (most importantly, they found greater resolution within Pseudosuchia). These differences, along with a slight difference in tree length of the recovered most parsimonious trees, is probably due to the choice of outgroup taxa, as Franca et al. [110] constrained *Erythrosuchus*, *Euparkeria*, and Proterochampsidae as successive outgroups to Archosauria whereas we used *Erythrosuchus* as a single outgroup. Some of the differences, however, may be due to the realistic fact that different search strategies may recover different results when analyzing datasets with extreme amounts of homoplasy, as is the case with the Brusatte et al. [39] dataset.

#### Brusatte et al. [39] revised dataset

**Figure 14:**
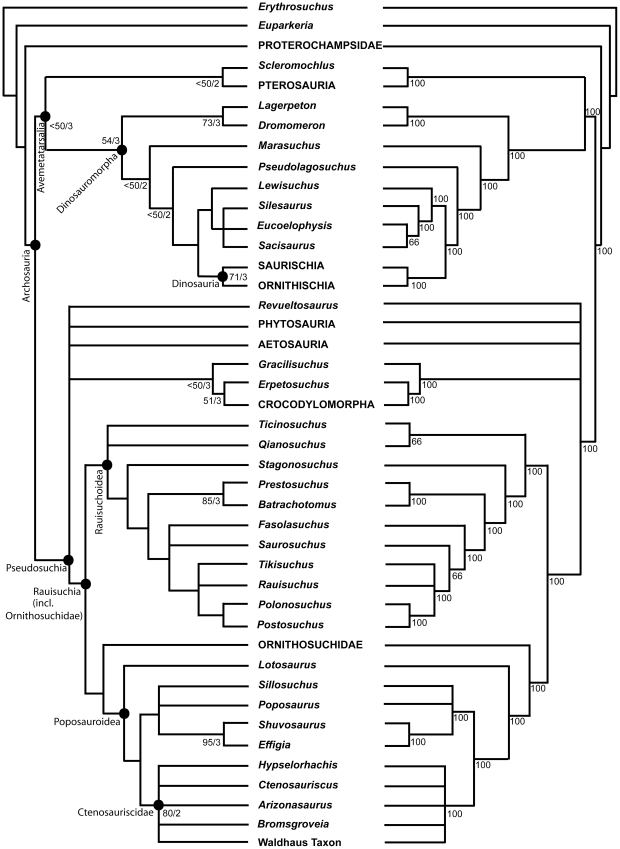
Strict consensus (left) and 50% majority rule consensus (right) resulting from the revised analysis of the Brusatte et al. [**39**] dataset. This dataset includes additional taxa (*Ctenosauriscus*, *Hypselorhachis*, Waldshut taxon) and characters (see text for details and search parameters). On the strict consensus, numbers next to clades denote Bremer/bootstrap values, the latter calculated with 1000 replications. On the majority rule consensus, numbers next to clades denote the percentage of most parsimonious trees in which that clade is recovered. All clades without support values have a bootstrap percentage of less than 50% and a Bremer support of 1 (i.e., they fall apart in the strict consensus of all MPTs and trees one step longer than the most parsimonious trees).

Analysis of the revised Brusatte et al. [39] dataset initially recovered 68 most parsimonious trees in the new technology search and a final set of 720 trees when these initial trees were subjected to TBR (tree length = 734; consistency index = 0.314; retention index = 0.691). The strict consensus and majority rule consensus of the 720 MPTs is shown in Figure 14. The strict consensus is substantially more resolved than the strict consensus recovered during the reanalysis of the original Brusatte et al. [39] dataset (Figure 13). Most importantly, a monophyletic Rauisuchia (including Ornithosuchidae) is recovered, as is a monophyletic Poposauroidea (including *Effigia*, *Shuvosaurus*, *Poposaurus*, and close relatives) and a monophyletic Rauisuchoidea (including large-bodied rauisuchians such as *Postosuchus*, *Prestosuchus*, *Saurosuchus*, and *Polonosuchus*). Both *Ctenosauriscus* and the Waldhaus taxon fall within a monophyletic Ctenosauriscidae, which also includes *Arizonasaurus*, *Bromsgroveia*, and *Hypselorhachis*. The high-spined *Lotosaurus*, on the other hand, is recovered as the basal-most poposauroid and outside of the ctenosauriscid clade. Relationships within Ctenosauriscidae are unresolved, but importantly, the clade has relatively high support values (bootstrap = 80%; Bremer support = 2). Support values for most clades are extremely low, due to high amounts of homoplasy, but Ctenosauriscidae stands out as one of the best supported clades in the phylogeny.

#### Nesbitt [6] dataset

**Figure 15:**
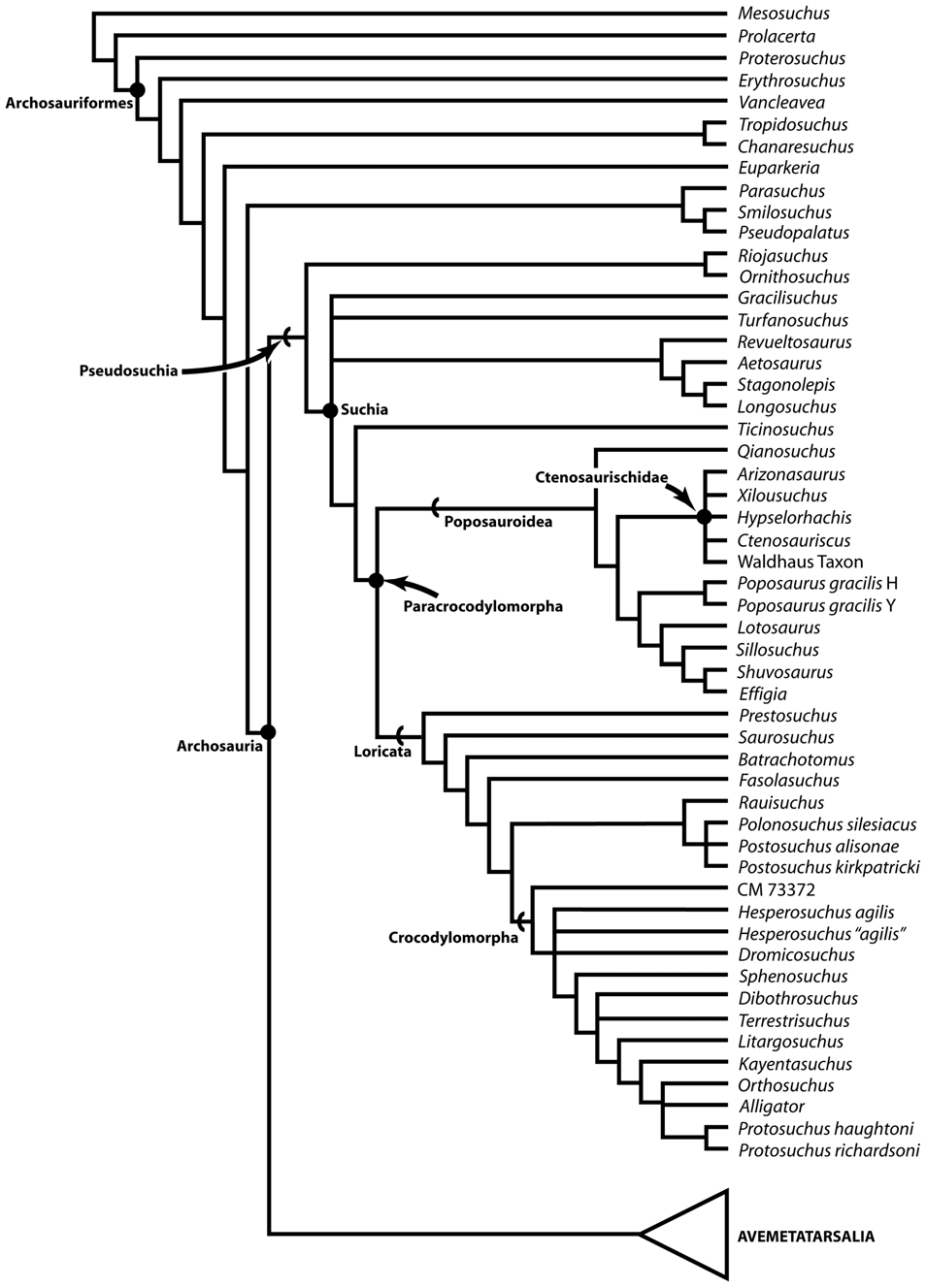
Strict consensus resulting from the revised dataset of Nesbitt [**6**]. Strict consensus generated from 360 MPTs (tree length = 1294; consistency index = 0.376; retention index = 0.776).

Our analysis of the modified Nesbitt [6] dataset recovered 360 MPTs (tree length = 1294; consistency index = 0.376; retention index = 0.776) with a nearly identical topology to that of Nesbitt [6] (Fig. 15). All of the hypothesized ctenosauriscid taxa in the dataset (*Ctenosauriscus*, the Waldhaus taxon, *Hypselorhachis, Arizonasaurus,* and *Xilousuchus*) were found in a completely unresolved monophyletic group near the base of Poposauroidea. The other sail-backed poposauroid, *Lotosaurus* was found closer to Shuvosauridae than Ctenosauriscidae, as in Nesbitt [6]. Ctenosauriscidae is relatively well supported (bootstrap = 57%; Bremer support = 2).

## Discussion

### Monophyly of Ctenosauriscidae

In the revised version of the Brusatte et al. [39] analysis, two characters unambiguously support the monophyly of Ctenosauriscidae, and another four characters unite the clade in many most parsimonious trees. The unambiguous characters include dorsal neural spines that are more than seven times taller than centrum height (character 84, state 2) and strongly curved dorsal neural spines (character 191, state 1). The ambiguous characters include: an ilium with a C-shaped articulation for the first cervical rib (character 95, state 2; also seen in *Batrachotomus*, basal crocodylomorphs, and dinosauromorphs); a reversal to a fully closed acetabulum (character 113, state 0; also seen in many other non-dinosauromorph and poposauroid taxa); and cervical neural spines with a dorsal margin that is greater than 150% of the anteroposterior length of its greatest anteroposterior constriction (character 189, state 1; also present in *Qianosuchus*). The final ambiguous synapomorphy, the possession of a small, shallow, and finger-like preacetabular process of the ilium (character 117, state 1), is scored as present in *Arizonasaurus*, *Bromsgroveia*, and the Waldhaus taxon, but it cannot be assessed in *Ctenosauriscus* and *Hypselorhachis* because ilia are unknown for these taxa. Therefore, the derived state of this character could either unite Ctenosauriscidae itself, or a less inclusive clade within Ctenosauriscidae including *Arizonasaurus*, *Bromsgroveia*, and the Waldhaus taxon.

In the modified version of the Nesbitt [6] analysis, Ctenosauriscidae is supported by four unambiguous characters: the presence of a parabasisphenoid plate that is arched anteriorly (character 96, state 1, uncertain for all taxa other than *Arizonasaurus* and *Xilousuchus*); posterior cervical vertebrae with neural spines that arc anteriorly (character 194, state 1); dorsal vertebrae with neural spines >4 times taller than the neural spines of the posterior cervical vertebrae (character 198, state 2); dorsal vertebrae with neural spines that are strongly curved, and which extend several centimetres beyond the anterior or posterior margin of the centrum (character 415, state 1).

Both analyses recover a monophyletic Ctenosauriscidae that excludes *Lotosaurus*, but includes *Arizonasaurus*, *Xilousuchus*, *Ctenosauriscus*, the Waldhaus taxon, and *Hypselorhachis*. The most consistent characters supportining Ctenosauriscidae are the great elongation of the dorsal neural spines, and the fact that these elongated neural spines are curved along their length.

### Function of the neural spines of *Ctenosauriscus*

The function of the elongated neural spines of *Ctenosauriscus* has been the subject of only a brief study by Ebel (in [31]), who developed biomechanical hypotheses of neural spine function. Using the skeleton of a moose (*Alces alces*) as a model, he argued that the elongated neural spines of the pectoral region of large mammals serve as levers to reduce subhorizontal tensile and counteracting compressive forces induced by the mass of the head and anterior trunk and to transmit these stresses through the forelimb into the ground. According to this model the arcuate arrangement of the neural spines reflects the necessity for optimal step-wise orientation of the neural spine axes relative to the force-transmitting forelimb during its rotation in a parasagittal plane at the shoulder-joint.

In a second step, Ebel (in [31]) applied these basic considerations to the biomechanics of *Ctenosauriscus*. Reconstructing force vectors originating from the tips of the neural spines, based upon the reconstruction of the vertebral column by Krebs [30] (see [31]: fig. 6), he found that most of these vectors met at a single point below the dorsal vertebral column. In this location he assumed the position of the knee-joint, and argued that the dorsal neural spines acted to absorb the ground reaction force transmitted from the foot and ankle through the zeugopodium during a step-cycle. By placing the hypothetical position of the knee-joint on a life reconstruction, Ebel concluded that *Ctenosauriscus* was at least facultatively bipedal ([31]: fig. 7).

However, although we agree that the elongated neural spines may well have had a biomechanical function, we identify a number of problems with the arguments and conclusions of Ebel. Most importantly, the transmission of forces requires a direct physical connection of the relevant elements that is in line with the internal force vectors. In the case of the shoulder-joint, this connection is provided by the trapezoidal and rhomboidal musculature, linking the scapula with the pectoral neural spines. However, there is no direct physical connection between the knee-joint and the mid-dorsal neural spines along any inferred force vector during hindlimb movement. By contrast to the pectoral girdle, the pelvic girdle is fixed relative to the vertebral column and forms a rigid, transversely arcuate, force-absorbing structure between the acetabula. The external ground reaction force in the hindlimb is transmitted from the autopodium to the acetabulum and via the sacral ribs to the sacral centra (e.g. [111]). The protracted, flexed position of the knee-joint in the figure of Ebel ([31]: fig. 7) represents a metastable state, in which the line of action of the internal ground reaction force through the limb is controlled by muscular force input and actuation. These fundamental differences in the construction of the tetrapod pectoral and pelvic girdle result in the fact that there is no direct force-transmissive connection between the dorsal neural spines and the hindlimb weight-bearing apparatus. There is neither a deviation of the external ground reaction force vector from pointing to the acetabulum in any stage of the hindlimb movement, nor a transmission of the external ground reaction force through the knee-joint into the dorsal neural spines.

We therefore consider the conclusion of Ebel (in [31]) that *Ctenosauriscus* was habitually bipedal as unsubstantiated on biomechanical grounds. The appendicular skeleton in other, more completely known ctenosauriscids, especially *Arizonasaurus,* does not provide evidence of bipedal locomotion in this group. What is known of the ctenosauriscid girdle and limb skeletons is comparable to other early pseudosuchian archosaurs exhibiting semi- to fully-erect quadrupedal stances and gaits (e.g. *Postosuchus*, *Ticinosuchus*, *Batrachotomus*). In fact, the weight added by the elongated neural spines in *Ctenosauriscus* likely resulted in an anterior shift of the center of mass, which is unfavorable for an elevation of the anterior body and bipedal locomotion. The only known clade of bipedal tetrapods with an anteriorly displaced center of mass is birds. In this group, the feet are located far anteriorly in order to place them below the centre of mass (e.g. [112]). This shift requires numerous modifications of the pelvis and hindlimb skeleton (especially the horizontally positioned femur [113]), which are generally absent in basal pseudosuchian archosaurs. Additionally, bipedal archosaurs typically show a trend of expansion of the preacetabular process of the ilium in anterior and either transverse (e.g. many ornithischians) or, more commonly, dorsoventral directions (e.g. in theropods and basal sauropodomorphs). Interestingly, derived, bipedal poposauroids (shuvosaurids) convergently show a similar iliac expansion resembling that of derived theropods [98], [114], suggesting that the homoplastic development of this feature was controlled by biomechanical advantages linked to bipedal, cursorial locomotion. However, this expansion is absent in more basal poposauroids, including ctenosauriscids.

In conclusion, it seems reasonable and most parsimonious to consider *Ctenosauriscus* as an obligatory quadruped, as also inferred for *Arizonasaurus* [18] and most other pseudosuchians. A more detailed analysis of the biomechanical function of the vertebral column in *Ctenosauriscus* is beyond the scope of this work.

### Stratigraphic and palaeobiogeographical distribution of the earliest archosaur body fossils (Olenekian–Anisian)

#### China

*Xilousuchus sapingensis* from the Heshanggou Formation of the Ordos Basin, Shaanxi Province, China was recently identified as the oldest archosaur by Nesbitt et al. [11] and Nesbitt [6]. *Xilousuchus* was originally identified as a proterosuchid [74], and later as an erythrosuchid [115], but Nesbitt et al. [11] and Nesbitt [6] reidentified it as a ctenosauriscid poposauroid archosaur closely related to *Arizonasaurus*. Radioisotopic, magnetostratigraphic, and invertebrate biostratigraphic data are currently not available for the Heshanggou Formation, and the age of the formation is inferred from palynomorph, macroplant and vertebrate biostratigraphy.

Shu & Norris [116] described palynomorphs from the upper part of the Heshanggou Formation, on the basis of which they correlated the unit with the Upper Buntsandstein (Röt Formation) of Germany and the “Waterstones Formation” of England ( =  Tarporley Siltstone and Bromsgrove Sandstone formations [117], [118]), and assigned it an Early Triassic (Olenekian) age. However, the Upper Buntsandstein [43]–[45], [56] and the Tarporley Siltstone and Bromsgrove Sandstone formations [117], [118] are considered Anisian in age, the lattermost yielding the *Arizonasaurus*-like poposauroid *Bromsgroveia* [16], [118]. Shu & Norris [116] also noted that the Heshanggou Formation has yielded a *Pleuromeia sternbergii* macroplant assemblage; in Germany, *Pleuromeia sternbergii* occurs in the Hardegsen Formation (late Olenekian) and throughout the Solling Formation (late Olenekian–earliest Anisian), including the uppermost Chirotherien-Sandstein of Thuringia [119], [120], recognised as earliest Anisian (Aegean) on the basis of conchostracan and palynomorph biostratigraphy and palaeomagnetic data [43]–[48]. Thus, the palynomorph and macroplant remains are consistent with an Anisian age for at least the uppermost parts of the Heshanggou Formation.

Tetrapod-based biostratigraphic correlations for the Heshanggou Formation have been attempted by a number of authors. The vertebrate assemblage comes from the upper part of the Heshanggou Formation [121], [122] and has been argued to be of ‘Lootsbergian’ (earliest Triassic) age “based primarily on the procolophonids” ([123]:454), although the rational for this assignment is unclear, particularly given that the Heshanggou procolophonids, *Eumetabolodon bathycephalus* and *Pentaedrusaurus ordosianus*, are closely related to both Olenekian and Anisian taxa [124]. Rubidge [125] correlated the ‘lower’ Heshanggou assemblage with Subzone A of the *Cynognathus* Assemblage Zone of South Africa (late Olenekian) and the ‘upper’ Heshanggou assemblage with Subzone B (early Anisian) of the *Cynognathus* Assemblage Zone, the latter based upon the apparent shared present of the dicynodont *Kannemeyeria*. At present no dicynodont has been described from the Heshanggou Formation [126], although Nesbitt et al. [11] listed *Shaanbeikannemeyeria xilougouensis* (IVPP V11675, a subjective junior synonym of *Kannemeyeria* [125], [126]) following Cheng [127]. Moreover, Rubidge [125] did not specify which members of the Heshanggou Formation vertebrate assemblage came from the ‘lower’ and ‘upper’ parts; in fact, the detailed stratigraphic distribution of vertebrates within the Heshanggou Formation has not yet been described in detail, although Nesbitt et al. [11] note that *Xilousuchus* comes from the ‘lower’ assemblage rather than the ‘upper’ assemblage that includes *Shaanbeikannemeyeria*.

Finally, Nesbitt et al. [11] noted that an undescribed taxon very similar to *Proterosuchus fergusi* occurs in the Heshanggou Formation and that this may support an Early Triassic age. *Proterosuchus fergusi* is known from the *Lystrosaurus* Assemblage Zone of South Africa and is an index fossil of the Lootsbergian Land Vertebrate Faunachron [122], [123]. *Chamatosaurus yuani* from the Jiucaiyuan Formation of China has been often mentioned as synonymous with *Proterosuchus* [122], [123] although no formal taxonomic revision has been carried out. However, a species of *Chasmatosaurus*, *C. ultimus*, has also been named from the late Anisian upper Ermaying Formation of China [115], [128], indicating that *Proterosuchus*-like taxa persisted into the Middle Triassic.

In summary, the palynological evidence suggests an early Anisian age for at least the uppermost part of the Heshanggou Formation, and this is supported by the presence of the dicynodont *Kannemeyeria*. Macroplant remains can only constrain the formation to late Olenekian–early Anisian. The strongest evidence for an Early Triassic age for at least the lower assemblage of the Heshanggou Formation is the presence of *Proterosuchus* [11], although as discussed above, *Proterosuchus*-like taxa survived into the Anisian in China, and also in Russia [7], [129]. Further work is needed to precisely constrain the age of the Heshanggou Formation and the distribution of vertebrates within it, but a Lootsbergian assignment seems unlikely (*contra* [123]), at least for the entire formation, and at present we conservatively consider *Xilousuchus* to be late Olenekian–early Anisian in age, of a broadly similar age to *Ctenosauriscus*.

#### Russia

Abundant, but generally disarticulated and fragmentary archosauriform material has been described from the Lower and Middle Triassic of European Russia [7]. The oldest material currently considered as belonging to crown Archosauria from Russia, and the only from the Early Triassic, is *Vytshegdosuchus zbeshartensis* from the Yarenskian Gorizont [130], which possesses one character of the ilium (a rugose ridge dorsal to the acetabular rim) that suggests referral to this group [6], [7], [11]. Nesbitt [6] recovered *Vytshegdosuchus* as a paracrocodylomorph using a numerical phylogenetic analysis. The Yarenskian Gorizont is generally considered late Olenekian in age on the basis of palynology and the presence of the characteristic Olenekian temnospondyl *Parotosuchus* [130]. In Germany, *Parotosuchus* occurs within the Volpriehausen, Hardegsen and lower Solling formations of the Middle Buntsandstein, and is therefore broadly of Olenekian age and considered an index taxon for the Nonesian [56], [123], [131]. *Vytshegdosuchus* is from the upper part (upper biochron: Gamskian) of the Yarenskian Gorizont and may therefore be latest Olenekian in age and approximately contemporaneous with *Ctenosauriscus*.

#### USA

The ctenosauriscid archosaur *Arizonasaurus babbitti* and an unnamed poposauroid have been collected from the Holbrook and Anton Chico members of the Moenkopi Formation of Arizona and New Mexico [3], [18], [19], [132]. The Holbrook and Anton Chico members have been assigned to the early Anisian (Aegean–Bithynian) based upon magnetostratigraphy and the temnospondyl *Eocyclotosaurus*, which occurs in the Upper Buntsandstein (Röt Formation) of Germany [56], with Kozur & Weems [47] arguing for a Bithynian age based on conchostracans.

#### Elsewhere

Archosaur material of broadly Anisian age is known from the Bromsgrove Sandstone and Otter Sandstone formations of England [16], [24], [101], the Donguz Svita of Russia [7], the Yerrapalli Formation of India [20] and the upper Ermaying, Xinlingzehn, Guanling and Kelamayi formations of China [6], [15], [21], [133]. The abundant and diverse archosaur assemblage from the Lifua Member of the Manda Beds of Tanzania [12], [13], [22], [23], [25] has been correlated to Subzone C of the *Cynognathus* Assemblage Zone of South Africa of late Anisian age [23], [134].

### Timing of the archosaur radiation

**Figure 16:**
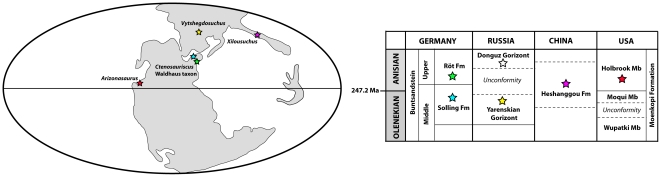
Geographic and stratigraphic distribution of the earliest archosaurs. Early Triassic palaeogeographical map (left) showing the distribution of the earliest known archosaur body fossils. Stratigraphic correlations (right) between formations yielding the earliest known archosaur body fossils.

The above review suggests that no unambiguous crown archosaur body fossils are known from prior to the latest Olenekian. The oldest archosaur body fossils appear to be *Vytshegdosuchus* from Russia and *Ctenosauriscus* from Germany, both latest Olenekian in age, and possibly *Xilousuchus* from China, of late Olenekian or early Anisian age (Fig. 16). Slightly younger appear to be *Arizonasaurus* from the Holbrook Member of the Moenkopi Formation of the USA and the ctenosauriscid material from the Röt Formation of Baden-Württemberg, both of earliest Anisian age (other Anisian archosaur faunas mentioned above are either dated as late Anisian, or lack well-constrained ages). Thus, all of these very early archosaur body fossil records are probably from a relatively short period of time, approximately coincident with the Early Triassic/Middle Triassic (Olenekian/Anisian) boundary. Current radioisotopic dates for the Early Triassic/Middle Triassic boundary place it at 247.2 Ma [2] and suggest a date of 252.3 Ma for the base of the Triassic. Thus, the first crown archosaur body fossils appear in the fossil record 4–5 million years after the Permian/Triassic extinction event. Less than 10 million years after the Permian/Triassic extinction event, during the late Anisian (the Anisian/Ladinian boundary is dated at 242 Ma [2]), highly diverse archosaur faunas were present in at least some parts of the world (e.g. the Manda Beds of Tanzania [12], [13], [22], [23], [25]).

As discussed by Nesbitt et al. [11] and Nesbitt [6], the relatively derived phylogenetic positions of *Ctenosauriscus* and *Xilousuchus* within Pseudosuchia imply that a large number of archosaur ghost lineages must extend back into at least the late Early Triassic, including Avemetatarsalia (‘bird-line’ archosaurs), Aetosauria, Ornithosuchidae, and a number of basal paracrocodylomorph lineages. Moreover, the earliest members of the lineages leading to a number of non-archosaurian archosauriform groups, including Phytosauria, Proterochampsidae, *Vancleavea*, Doswelliidae, and Proterochampsidae, must also have been present by the late Early Triassic [6], [11]. However, with the possible exception of the putative Early Triassic phytosaur *Mesorhinosuchus* (the holotype of which is lost and the locality data controversial [135]) and the paracrocodylomorph *Vytshegdosuchus*, no Early Triassic body fossil evidence for any of these lineages has yet been identified. Thus, a large number of Early Triassic archosaur and archosauriform lineages remain unsampled by palaeontologists, implying that the early archosaur record is still highly incomplete [6], [39]. Even more striking is the decreasing temporal distance between the inferred onset of the crown archosaur radiation and the Permian/Triassic mass extinction event – current evidence would suggest either a very rapid origin and radiation of the archosaur crown group in the late Olenekian or, perhaps more likely, an origin earlier in the Early Triassic, in the immediate aftermath of the extinction. Although a Permian origin for crown Archosauria cannot be discounted, there is no direct body fossil or ichnological evidence at present to support this hypothesis.

Brusatte et al. [9] reached a similar conclusion based upon the ichnological record: they described early–late Olenekian footprints from Poland that they assigned on the basis of synapomorphies to the dinosauromorph lineage; these footprints suggest an initial radiation of archosaurs including dinosauromorphs by the early Olenekian, perhaps within just two million years of the Permian/Triassic extinction. Further detailed study of Lower Triassic footprint assemblages may shed new light on the timing of the archosaur radiation.

Intriguingly, all of the earliest crown archosaur records currently known (i.e. those clustering around the Olenekian/Anisian boundary), with the probable exception of the poorly known *Vytshegdosuchus* [6], are of ctenosauriscids, which are geographically widespread within the northern hemisphere at this time (China, central Europe, western USA: Fig. 16). Nesbitt [6] also noted that poposauroids, the clade that includes ctenosauriscids, are the most commonly recovered early archosaur fossils. Butler et al. [22] tentatively suggested that the apparent restriction of ctenosauriscids to the Anisian might make them useful for biostratigraphical purposes (i.e. identifying Anisian-age deposits), but this suggestion is partially contradicted by evidence here that demonstrates a minimum late Olenekian–late Anisian range for the clade. One possible solution to the puzzle of the missing Early Triassic archosaur lineages is that the early radiations of these clades took place in geographical areas (e.g. the tropics [11]) or environments that are not well sampled in the Early Triassic fossil record. Under this hypothesis, ctenosauriscids may represent the first global radiation of archosaurs outside of these poorly sampled environments/geographical areas, although their success appears to have been relatively short-lived, with the clade perhaps surviving for less than 10 million years.

## Methods

### Phylogenetic analysis

#### Brusatte et al. [39] revised dataset

To analyze the phylogenetic position of the German material and assess ctenosauriscid monophyly, we performed a cladistic analysis using a modified version of the Brusatte et al. [39] dataset (see Text S1, Materials S1). We added six characters and three taxa (*Ctenosauriscus*, the Waldhaus taxon, *Hypselorhachis*), corrected a handful of erroneous scorings in the original analysis, and deleted a problematic character (character 80) (see Text S1 for a full description of the new data and character changes). The end result was a 58 taxon, 192 character dataset, which we analyzed in TNT using the search strategy outlined above. Several preliminary runs showed that two pseudosuchian taxa, *Arganasuchus* and *Yarasuchus*, were acting as wildcards, so we proceeded by deleting these taxa from the analysis. Bootstrap and Bremer supports were calculated using TNT, the former with 1000 replicates and the latter by saving topologies up to 10 steps longer than the minimum length.

#### Nesbitt [6] dataset

We also tested the monophyly of Ctenosauriscidae in the independent basal archosaur dataset of Nesbitt [6] (see Text S1, Materials S2). We added to this dataset three of the completely new characters also added to the dataset of Brusatte et al. [39] (Text S1: characters 413–415). Characters 188 and 192 added to Brusatte et al. [39] have already been incorporated as characters 194 and 273, respectively, in the dataset of Nesbitt [6]. A third state was added to character 198 of the dataset of Nesbitt [6] and this character was treated as ordered. Three taxa (*Ctenosauriscus*, the Waldhaus taxon, *Hypselorhachis*) were added to the dataset of Nesbitt [6], but *Bromesgroveia* was not added. The end result was an 81 taxon, 415 character dataset, which we analyzed in TNT using the search strategy described in Nesbitt ([6]:184). The ordering of characters was identical to that given in Nesbitt ([6]:188) with the addition of treating modified character 198 as ordered. Bootstrap and Bremer supports were calculated using TNT (100 replicates).

## Supporting Information

Text S1 **Additional information on the phylogenetic analysis. Includes new characters and taxon scores.**
(DOC)

Materials S1 **Data matrix for the revised analysis of the Brusatte et al.** [**39**] **dataset.**
(TXT)

Materials S2 **Data matrix for the revised analysis of the Nesbitt** [**6**] **dataset.**
(NEX)
