# Supplementary material for: The Sail-Backed Reptile Ctenosauriscus from the Latest Early Triassic of Germany and the Timing and Biogeography of the Early Archosaur Radiation
Source: PLoS One. 2011 Oct 14;6(10):e25693. doi: 10.1371/journal.pone.0025693 (PMC3194824; doi:10.1371/journal.pone.0025693)
Supplement: Text S1 — Additional information on the phylogenetic analysis. Includes new characters and taxon scores. (DOC) [file pone.0025693.s001.doc]

Supplementary Material to:

**The Sail-Backed Reptile *Ctenosauriscus* from the Latest Early Triassic of Germany and the Timing and Biogeography of the Early Archosaur Radiation**

**Richard J. Butler, Stephen L. Brusatte, Mike Reich, Sterling J. Nesbitt, Rainer R. Schoch, Jahn J. Hornung**

**1. Additions and Corrections to the Brusatte et al. (2010) dataset.**

**Character Revisions:**

1) Character 80 is deleted (but it remains in the data matrix below), because it is difficult to score this character (which relates to the constriction of the cervical vertebral centra) consistently on the same, or similarly positioned, vertebrae in the cervical column across all taxa.

2) Character 113, regarding a perforate acetabulum, is divided into two characters. Character 113 remains as a character distinguishing between a closed, incipiently open, and fully open acetabulum, whereas a new character (192) refers specifically to a concave ventral margin of the ischial peduncle. Basal poposauroids (*Arizonasaurus*, *Bromsgroveia*, Waldshut taxon) are not scored as possessing an incipiently open acetabulum for character 113, but are scored for possessing a concave ventral margin of the ischial peduncle for character 192.

3) Character 84 is changed into an ordered multistate, with the addition of a new second derived state describing dorsal neural spines greater than 7 times centrum height. This second derived state is present in *Arizonasaurus* and *Ctenosauriscus*, *Hypselorhachis*, and the Waldshut taxon.

**Scoring Changes**:

*Arizonasaurus*: character 119, 0 to 1

*Bromsgroveia*: character 112, 1 to 0; character 120, 1 to 0.

*Effigia*: character 79, 1 to 0.

*Polonosuchus*: All positive scores for ilium characters (characters 110-121) are changed to “?” (we no longer consider a referred ilium to represent a specimen of *Teratosaurus* or *Polonosuchus*: Brusatte et al. 2009).

*Poposaurus*: character 3, 0 to ?; character, 12 0 to ?.

**New Characters**:

188) Cervical vertebrae, orientation of neural spines in middle-posterior cervicals: projecting straight dorsally or slightly posteriorly (0); curving anteriorly (1). State 1 is present in *Arizonasaurus*, *Ctenosauriscus*, *Lotosaurus*, and *Xilosuchus*.

189) Cervical vertebrae, shape of neural spines of middle-posterior cervicals: dorsal margin less than (0) or greater than (1) 150% anteroposterior length of strongest anteroposterior constriction. State 1 is present in *Arizonasaurus*, *Ctenosauriscus*, and *Xilosuchus* (not included in this analysis), as well as *Qianosuchus*. State 0 is present in *Lotosaurus*.

190) Dorsal vertebrae, shape of neural spines in those taxa with elongated spines, dorsal margin less than (0) or greater than (1) 200% anteroposterior length of strongest anteroposterior constriction. State 0 is present in *Arizonasaurus*, *Ctenosauriscus*, *Hypselorhachis*, and the Waldshut taxon. State 1 is present in *Lotosaurus* and currently optimizes as an autapomorphy of this taxon, but is included here in case future discoveries show that it is a synapomorphy of a less inclusive clade.

191) Dorsal vertebrae, curvature of neural spines (on anterior and posterior dorsals): weakly curved, spines stop short of extending past the centrum or marginally extend pass the level of the centrum (0); strongly curved, spines extend several centimeters past centrum (1). State 1 is present in *Arizonasaurus*, *Ctenosauriscus*, *Hypselorhachis*, and the Waldshut taxon. State 0 is present in *Lotosaurus*.

192) Ilium, form of the ventral margin of the ischial peduncle: straight or convex (0); concave (1). Note that the “concave” condition is equivalent to the “incipiently open acetabulum” condition that is often used in phylogenetic analyses and is often optimized as a poposauroid synapomorphy. Some derived poposauroids do possess an acetabulum that is truly incipiently open as a small slit, but basal taxa such as *Arizonasaurus* do not. Rather, the unusual condition of all or most poposauroids is that the ischial peduncle has a concave ventral margin. This condition is associated with another unusual feature of the pelvis in some of these taxa (clearly *Arizonasaurus*): the pubis and ischium do not make contact, but rather are separated from each other by the acetabular plate of the ilium (see Nesbitt 2005 for description).

193) Ilium, placement of ridge extending from the dorsal margin of the acetabulum relative to the notch between the preacetabular process and pubic peduncle: posterior, smooth margin of bone between ridge and notch (0); anterior, ridge abuts notch (such that, if present, the fossa located anterior to the ridge faces anteriorly only, and not at all laterally) (1). State 1 is present in *Arizonasaurus* and *Bromsgroveia*.

**Revised TNT Matrix:**

xread

193 58

Erythrosuchus0001000000000210010101000000100000000100010000?00000000000000?00?000?0010010000000000010000?001??00000??0011?00000?000000000000000000000?000?00??000000?00?00001?000001000000000000?010000?00?00?

Euparkeria000001001000021000000100000110010000000000000000000000000000000000000000?01000000000100000000000000000000000?00000?0000000000000000000?0?0000001000000?000??000000000000000000000000000000000?00?

PROTEROCHAMPSIDAE1000000000000001000000100000000000020000000000000010000000100???0?????00000000000000000000000001000000000000?00000?0000000000000000000?0????000000?000??00?00000000000000000000?0000001000000?00?

Scleromochlus101002?0100000?000?0110000001??00000?0?001000???00?2?00????????1??????01?????000???0???20?00??02?100??110000?0?000???1?0001???00010000?0?????00?1??100??01?0000??0?00???0?????1?0111011100000?00?

Eudimorphodon10001000100000010010000000?0?0000?0?????00000???0??0??????????????????01????01000??0000200000??2?00000110001001100?1010000000000000000?0????0??010?10100?0?2010???0???????1???1?011?01??00000?00?

Dimorphodon10001000?00000010010000000?0?0??0?00????00000??????0??????????????????0010??1100000000?20000?002?00000110000001100?00100000000000000000001?0000010?101000002010???0???????1???100111011000000?00?

Pteranodon1000??000?00?0??10100001000010100?002??0000000?00?020001100???0???0???20100111000000000210000002?00000110011001000?1010000000000000000?0????000010?101000002010???0????1??1???110111010101000?00?

Lagerpeton???????????????????????????????????????????????????????????????????????????????????00??00???0112?????????????00100?00000010001010110010001?1000001?10?0101020200?1100101121???1011101011110???00?

Dromomeron??????????????????????????????????????????????????????????????????????????????????????????????????????????????????????????????????10010001?0000011111??1010??20??1100101021??????????????????????

Marasuchus?0????????0???????00?1?0???????????????????????????????????00021?1??????????112000000000000?0012?10000110001?10100?0000001200210010000010[1,2]?1111000111?1001120000?1000101101000101110101111?00?00?

Pseudolagosuchus???????????????????????????????????????????????????????????????????????????????????????0??0????2?????????????????????????1?0020???00000021?111110??11?1001120001?200010?101000???????????????????

Silesaurus00?001????0?021000000100000?1??0000?????0?0????????0000???00002101???010000111200000010100001022?01001110000?10100?00002012002001101001122?111110010211011000001?2000111121??????010111011000?00?

Sacisaurus?0????????0??????0001100???????????????????00???????????0?????????????100??????0???000????0????2??10???????????100?00000?2?002??1?0100112[1,2]?1?10100??2110111??????2????????????????????????????00?

Eucoelophysis?????????????????????????????????????????????????????????????????????????????????????????????????????????????????????????2?0020???01001?22?1110100??1??????????????????????????????????0?????????

Lewisuchus???????0??????????0011?0????????????????0?000?0?00001?0???0000?10?????00????1120000000?????0???2??1000??0000??????????????????????0001?????????1???0??????????????????????????????1???0???000?0??

Herrerasaurus0000010101000110000001000001100000120101000000000010000100100021011???00001?112010001111000?0022?10???110011110020?10000022002211102100102?111110001211011100001?2001111121???111010111011000?00?

Coelophysis000001000100010101[0,1]0110000011010001221010[0,1]0000000000?0010?1000?101????00000?11201010111211000022?10001100011110020?00102012002101102100112?11111001121101112010??2001?11??1????11010111011000?00?

Plateosaurus0000110001000101001011001001100000122100000000000000000100100021011??101000111001000011100000022?11001110011100020?0000202200210020210011[1,2]?111010000211011120001?2001111121???111010001011000?00?

Lesothosaurus0010110001010110000?1100000010000012210100000000000000010?1000?1011??000000111201000000200001012?110??110011111020?10102022002001101100112?11101001120101112000??2??1???121??????0101110??000?00?

Psittacosaurus0?10?2000100001?100???0000001000001221110?0000000000?001??100?????????000101000010000?0200001022?10000110011101020?1010202200200110210?1???1?10100?120??11?00001?200100?021???011010111011000?00?

Heterodontosaurus0010110001010110000?10000000100000122??1000000100000000???100??1??????01010?0120100000?20000??22?11?00110011111020?1010202200200110210?????1?10?00?120?0?1?2010???????????1???0?11101110?1000?00?

Paleorhinus100002000000110000001000000000000000010000000000000000000?000010000?0001110000000000100000?10?00100100000100000100?0000000000000000000?0?????00000?0001000?11010?001000?0101100?0000001000000?00?

Rutiodon100002000000110000001000000000000000010000000000000000000?????????????011?0?00000000100000010000100101000100000000?0000000000000000000?0?????00000?000?000???01010010000010110????????1????00?00?

Mystriosuchus100002000000110000000000000000000000000000000010000000000?000010000?0001100100000000100????1???0100101000100000000?001000000000000000000000000000000?????????0?????????????????????????????00?00?

Aetosaurus000012112000010001101100000000000000011001000001000000?00?000??0?21???10010????00????0?0?0??0?10101110000100?10000?001000211?1000100000000000000000000?000?11010?001000?0101100?00000100000????0?

Stagonolepis000012112000010000001000000000000000011000000001002000000?0000?002111110010100000000100000010010101010000100?00000?000000211011001000000?00000000000001?01?11010?00100000101100?0000010000000?00?

Desmatosuchus0000121110000100100010000000000000000110000000010020000000000020021??11001010000000010?000010010101010000100?10000?00100021101100100000000000001001000100101101000010000010110??000001?000?00?00?

Ornithosuchus0000010101000000010011?00001011000000100000010000020000000000???0?????0010110?0000000001001?0010100011000100?00010?000000121020002000000100001000??01??001??102??????????101100?00000?1000000?00?

Riojasuchus010011010000000001001100100101100000010000000000002000000?0000?001????01100?00000000000100???0?0?0?011??0100?0?010?000000121020?0?0000?0?0??010000?0101001?11020?00100000101100?0000101000000?00?

Terrestrisuchus00000101100???????001110000110010002?000010010?200?1100001000?????1??101000?100000000000001100201010120?0000011010?0010001210200010000000??00000000100000000001010010000010111000011011010000?00?

Sphenosuchus00000201110002100000101000110001011220110100111200?11000011000200211110100000?000000???????1???0101002000000????????????????????????????????????????0010000???????????????????????1?0?10??000?0??

Protosuchus00000200000002100000111000000000001220110100101200?110000?100020021??100000000000000000000?100?0101102??0100001010?0010002210210030000?0??????0000?000?000?0?010?001000?010111??0011011010000?00?

Erpetosuchus?000000111000110000011100001000?0002211100001111000110000?00???0??????00?00?0?0000001??????????01?11110?0000???????????????????????????????????????????????????????????????????????????????00?0??

Gracilisuchus0010021121000210000011?00001010000001100010011010021100000000??00?????00000?010000001000000100?1100100??0100?00000?00000021?010001000000?00000010??0101000000010100100000101110??110001110000?00?

Qianosuchus0000110110000001011111000??0?0000?00????01000?00?020?0????????????????00?00?1100??00??00??00???00?110?000000??100??00100010??100020000?0???????????0????00???01??001?0?0010110???0?00?0000001?01?

Revuletosaurus?000???0000?0210010111?0000?011000000??00?000?0200?0??????????????????00????0000?0001??0???????01???00??0100?00000?000000?????????0000?0?????0000????????????010?0010000010110????????????????00?

Arizonasaurus0000110100????????11010000011??0000001000?00000?00?0?00011?000010000000000?010110102011111??1122??1?0000?????01002101010012102101200000010000000000????0???????????????????????????????????110111

Batrachotomus000111010100020?001111?011100000010101010101101010?00000??100020010001010000000000001111000?012?1?1111?00100?010011000000211021002000000100000000???00000001101??????????10110???00??0?000000??00

Bromsgroveia?????????????????????????????????????????????????????????????????????????????????????1?111??112??????????????010021010100???????1??00????????000???????????????????????????????????????????????11

Effigia00100101000122011010110100011000000201101000000000?2111010?110211?1??020111110000010011211??11?2?10011?010???10012010111112112311212101010110000?01?0110000000101001100001????000010111010100?010

Fasolasuchus00??01?????00100001111?0000???????????????????????????????????????????000??000000000111???0????10??????????????????????????1?2????0110000??001000010???????1101010010000010111?????????????00?0??

Lotosaurus0001110010012101100011?100001000010?0101000000?00020000???1000??01????21100000001001011110?11102?0????0000000100121000101?????????00000011?0000000100010000000101001?0?0010110?????????????101010

Poposaurus????????????????????????????????????????????????????????????????????????????000001100112110?1112?11?????0000?0101210011102210220120200101000000000000?1?000??01010011000010111????????????????010

Postosuchus000101011110011000001100011101?010000001001110110130?00001000120010??00100?00000000001100011001??01011??0100001001000000022102200200000010000000001000100001001010011000010111??00000?1000000?000

Prestosuchus?101110111000100000111?01110000000010101010110001010?00???100??0??0???000000????????1111001?00111?0111000100?01002?0000002??021002000000100000000000000000011010100100000101100000000010000????00

Rauisuchus??0??1??1????1000???1??0000????????0????0?1?10?000?????0?????????????????000000000001010001?001?1?1?1????????0100110000001?1020?????????????????????00?00001101010010000???????????????????00?000

Saurosuchus0100010111100110000011100001000100000101000110001010000000000020010000000???000000001010000100110?00?????????0100110000002?1021002000000?????00?00?00?1?00?11010100100000101110000000100000???000

Shuvosaurus00100101000121?1100?10110001110000020110100000000022111010111021111??020111111000010???211??1112??0001??1000?1001201011111211231121210101011000010100110000?001010011000010110?00010111010100?010

Sillosuchus????????????????????????????????????????????????????????????????????????????1001?11001?2110?11?2?????????????01?120?0?10?12102101?0000?0????000?10????????????????????????????????????????????010

Stagonosuchus???????????????????????????????????????????00??????????????????????????????001000000101000??001??????0??0100?01001?000000201021003??????????????????0??????????????????????????????????????00?000

Polonosuchus?001020111000100000011100101?1101?????????1?10100130000001????????????000???0???000??????????01?1????????????????????????????????????????????????????????????????????????????????????????????????

Ticinosuchus0?????????????????111100??????????????????????????????????????????????00????010000001000??11???1000?000000000?1000?00000010?0200?20000?0???????000?000??0??11010?001000?0101110?00000110?0000?000

Tikisuchus000?????1?1???????0011?0????????0?0?????0?0???10??20?????????120??0???01?000?????????????????????????????????????????????1?1010??????????????????????????????010?00??0??010111???????????????????

Yarasuchus0?????????1??2????1?01?0????????????????0?0??????0?0?1??????????????????????1???00000?10000?0?1?101?10??0011?01000?0000001?1020???0000?0??????000??0001000?????????????????????????????????0??000

Arganasuchus??????????????????10?1?0??????????????????????????????????????????????000??????0??0????????????????????????????????????????1??????00000000000100000000?????11????????????????????????????????????

Ctenosauriscus???????????????????????????????????????????????????????????????????????????????????20??11??0???2???????????????????????????????????????????????????????????????????????????????????????????1101??

WaldhausTaxon???????????????????????????????????????????????????????????????????????????????????2?111?????12??????????????010021010100????????????????????????????????????????????????????????????????????0110

Hypselorhachis???????????????????????????????????????????????????????????????????????????????????2?11??????????????????????????????????????????????????????????????????????????????????????????????????????01??

;

**2. Additions to the Nesbitt (2011) dataset**

**Modified characters:**

Character 84 of the dataset of Brusatte et al. (2010) is equivalent to Character 198 of the dataset of Nesbitt (2011), with the addition of character state 2 to read as follows:

198. Dorsal vertebrae, neural spines: about the same height as the posterior cervical vertebrae neural spines (0); 2–4 times taller than the posterior cervical vertebrae neural spines (1); >4 times taller than the posterior cervical vertebrae neural spines (2). [The character is now ordered].

**Characters added to the Brusatte et al. dataset that are already present in similar form in the dataset of Nesbitt (2011):**

Character 188 of the dataset of Brusatte et al. (2010) = Character 194 of the dataset of Nesbitt (2011).

Character 192 of the dataset of Brusatte et al. (2010) = Character 273 of the dataset of Nesbitt (2011).

**New characters added to the dataset of Nesbitt (2011)**:

413. Cervical vertebrae, shape of neural spines of middle–posterior cervicals: dorsal margin less than (0) or greater than (1) 150% of anteroposterior length of strongest anteroposterior constriction. (= character 189 of the modified dataset of Brusatte et al. [2010], see Appendix 1).

414. Dorsal vertebrae, shape of neural spines in those taxa with elongated spines, dorsal margin less than (0) or greater than (1) 200% of anteroposterior length of strongest anteroposterior constriction. (= character 190 of the modified dataset of Brusatte et al. [2010], see Appendix 1).

415. Dorsal vertebrae, curvature of neural spines (on anterior and posterior dorsals): weakly curved, spines stop short of extending past the centrum or marginally extend beyond the anterior/posterior margin of the centrum (0); strongly curved, spines extend several centimetres past anterior/posterior margin of centrum (1). (= character 191 of the modified dataset of Brusatte et al. [2010], see Appendix 1).

**Character scores for added taxa:**

*Ctenosauriscus* *koeneni* ???????????????????????????????????????????????????????????????????????????????????????????????????????????????????????????????????????????????????????????????????????????????????????????00000?1?002??1???001?????????????????????????????????????????????????????????????????????????????????????????????????????????????????????????????????????????????????????????????????????????????????????????????????????????????101

*Hypselorhachis* *mirabilis* ??????????????????????????????????????????????????????????????????????????????????????????????????????????????????????????????????????????????????????????????????????????????????????????????????1??20??????????????????????????????????????????????????????????????????????????????????????????????????????????????????????????????????????????????????????????????????????????????????????????????????????????????????????01

Waldhaus taxon ?????????????????????????????????????????????????????????????????????????????????????????????????????????????????????????????????????????????????????????????????????????????????????????????????????20????????????????????????????????????????????????????????????????01001000020000????????????????????????????????????????????????????????????????????????????????????????????????????????????????????????????????????????01

**Character scores for characters 413-415:**

*Mesosuchus browni* 0?0

*Prolacerta broomi* 0?0

*Proterosuchus fergusi*  0?0

*Erythrosuchus africanus* 0?0

*Vancleavea campi* 0?0

*Chanaresuchus bonapartei* 0?0

*Tropidosuchus romeri* 0?0

*Euparkeria* *capensis* 0?0

*Parasuchus* *hislopi* 0?0

*Smilosuchus* *gregorii* 0?0

*Pseudopalatus* *pristinus* 0?0

*Gracilisuchus* *stipanicicorum* 0?0

*Turfanosuchus* *dabanensis* ???

*Ornithosuchus* *longidens* 0?0

*Riojasuchus* *tenuisceps* 0?0

*Revueltosaurus* *callenderi* 0?0

*Aetosaurus* *ferratus* ???

*Stagonolepis* *robertsoni* 0?0

*Longosuchus* *meadei* 0?0

*Ticinosuchus* *ferox*  0?0

*Qianosuchus* *mixtus* 1?0

*Xilousuchus* *sapingensis* 10?

*Arizonasaurus* *babbitti* 101

*Poposaurus* *gracilis* holotype ???

*Poposaurus* *gracilis* Yale ??0

*Lotosaurus* *adentus* 010

*Sillosuchus* *longicervix* ??0

*Effigia* *okeeffeae* 1?0

*Shuvosaurus* *inexpectatus* ???

Combined *Prestosuchus* 0?0

*Saurosuchus* *galilei* 0?0

*Batrachotomus* *kuperferzellensis* 0?0

*Fasolasuchus* *tenax*  0?0

*Rauisuchus* *triradentes* 0?0

*Polonosuchus* *silesiacus* 0??

*Postosuchus* *kirkpatricki* 0?0

*Postosuchus* *alisonae* ??0

CM 73372 ???

*Hesperosuchus* *agilis* 0?0

*Dromicosuchus* *grallator* ???

*Hesperosuchus* “*agilis*” ???

*Dibothrosuchus* *elaphros* ???

*Terrestrisuchus* *gracilis* 0?0

*Sphenosuchus* *acutus* 0??

*Litargosuchus* *leptorhynchus* 0?0

*Kayentasuchus* *walkeri* ???

*Orthosuchus* *stormbergi* 0?0

*Alligator* *mississippiensis* 0?0

*Protosuchus* *haughtoni* 0?0

*Protosuchus* *richardsoni* ??0

*Eudimorphodon* *ranzii* ??0

*Dimorphodon* *macronyx* 0?0

*Lagerpeton* *chanarensis* ???

*Dromomeron* *gregorii* ???

*Dromomeron* *romeri* ???

*Marasuchus* *lilloensis* 0?0

*Asilisaurus* *kongwe* 0?0

*Eucoelophysis* *baldwini* ???

*Sacisaurus* *agudoensis* ???

*Lewisuchus*/*Pseudolagosuchus* 0?0

*Eocursor* *parvus* ???

*Silesaurus* *opolensis* 0?0

*Pisanosaurus* *mertii* ???

*Heterodontosaurus* *tucki* 0?0

*Lesothosaurus* *diagnosticus* ???

*Scutellosaurus* *lawleri* ???

*Herrerasaurus* *ischigualastensis* 0?0

*Staurikosaurus* *pricei* ??0

*Eoraptor* *lunensis* ??0

*Saturnalia* *tupiniquim* 0?0

*Plateosaurus* *engelhardti* 0?0

*Efraasia* *minor* 0?0

*Tawa* *hallae* 0?0

*Coelophysis* *bauri* 0?0

*Dilophosaurus* *wetherelli* 0?0

*Allosaurus* *fragilis* 0?0

*Velociraptor* *mongoliensis* 0?0
